# Economic evaluations of disease-modifying therapies for spinal muscular atrophy: a systematic literature review

**DOI:** 10.1186/s13023-025-04150-z

**Published:** 2025-12-05

**Authors:** Mehdi Yousefi, Amin Mehrabian, Anna Brown, Furqan Butt, Jeremiah Donoghue, Janette Parr, Mubarak Patel, Amy Grove, Jo Parsons, Peter Auguste

**Affiliations:** Centre for Evidence and Implementation Science, Health Services Management Centre, School of Social Policy and Society, College of Social Sciences, University of Birmingham, Birmingham, B15 2RT UK

**Keywords:** Systematic review, Economic evaluation, Health technology assessment, Spinal muscular atrophy, Cost-effectiveness, Rare diseases, Orphan diseases

## Abstract

**Background:**

Spinal muscular atrophy (SMA) is a rare, life-limiting neuromuscular disorder characterised by progressive motor neuron degeneration. The recent emergence of disease-modifying therapies (DMTs), nusinersen, onasemnogene abeparvovec, and risdiplam, has revolutionised SMA care but presents economic challenges due to high treatment costs and limited long-term evidence.

**Objective:**

To review and critically appraise economic evaluations that assessed the cost-effectiveness of DMTs in people living with SMA.

**Methods:**

A systematic literature review was conducted following Cochrane and PRISMA guidelines. Initial searches were conducted in January 2024 and updated in February 2025. Searches were carried out in key biomedical and economic databases, as well as grey literature. Two reviewers independently screened the titles and abstracts of all identified records, as well as the full texts of potentially relevant studies. Data extraction and quality appraisal employed established tools, including the CHEERS and Philips checklists. The conduct and findings of included studies were summarised and discussed narratively.

**Results:**

Of 1,984 records, 21 studies met the inclusion criteria. All studies used Markov modelling approaches, varying by SMA type, time horizon (often lifetime), and assumptions around sustained treatment benefits. Key drivers of cost-effectiveness included treatment costs, health-state utility values (frequently based on expert opinion), and survival modelling. Heterogeneity was noted in health technology definitions, utility measurement, and data sources. Limitations across studies included reliance on short-term clinical data, inconsistent assumptions, and limited of transparency in modelling practices. Sensitivity analyses were inconsistently applied, limiting robustness of the findings reported in each study.

**Conclusions:**

The economic evaluation landscape for SMA treatments is evolving. However, challenges remain due to data gaps and methodological variability across studies. Future research should prioritise the integration of long-term real-world data into economic evaluations, consider the development of patient- and caregiver-derived utility values, and the use of transparent, standardised modelling approaches. These improvements will enhance the robustness, comparability, and policy relevance of economic evaluations in rare disease treatment funding.

**Supplementary Information:**

The online version contains supplementary material available at 10.1186/s13023-025-04150-z.

## Background

Spinal muscular atrophy (SMA) is a rare genetic disorder and orphan disease characterised by the progressive degeneration of alpha motor neurons in the spinal cord, leading to muscle atrophy, weakness, and paralysis [1, 2]. The most common form of SMA arises from a deficiency in the survival motor neuron 1 (SMN1) gene, impacting approximately 1 in 11,000 births, SMA stands as a prominent genetic cause of infant mortality [1, 2].

SMA primarily affects motor neurons responsible for crucial functions like walking, crawling, and swallowing. The condition presents with varying symptoms and severity levels, leading to its classification into distinct types based on age at symptom onset and motor function proficiency [3, 4]. In the UK, the most prevalent classification of SMA, known as 5q SMA, presents with a clinical spectrum ranging from Type 1, manifesting in infants with severe impairment who cannot sit or roll independently, to Type 4, marked by relatively mild motor difficulties such as impaired walking in adulthood.[4].

It is also important to note that there is a Type 0 SMA, the rarest and most severe form, which presents prenatally or at birth with profound muscle weakness, respiratory failure, and minimal movement. Survival beyond a few weeks is uncommon, and treatments are generally considered ineffective due to the extreme severity and rapid progression of the disease [5, 6] Additionally, some studies have reported that presymptomatic SMA patients—identified through newborn screening or genetic testing—may benefit from early therapeutic intervention, although outcomes vary depending on the SMA subtype and the timing of treatment initiation.[7]

Historically, the management of SMA relied on supportive care approaches due to the unavailability of disease-modifying therapies (DMTs). This paucity of treatment prompted advancements in areas such as nutritional support and respiratory care, without the capacity to prevent motor neuron degeneration or enhance muscle strength [8, 9]. However, these non-pharmaceutical approaches did not alter the disease progression [10]. Subsequent updates to the standard of care (SoC) adopted a multidisciplinary framework, incorporating input from diverse healthcare professionals in anticipation of emerging therapeutic advancements[1, 5].

The evolution of the SMA treatment landscape saw the emergence of DMTs such as nusinersen, onasemnogene abeparvovec, and risdiplam, offering improved prognoses for individuals with SMA [10–13], each with distinct molecular approaches and administration.[14–16]

Clinical trials showed significant improvements in individuals with early- and later-onset SMA, reinforcing the benefits of early treatment initiation, particularly in the presymptomatic phase.[17–24]

Despite the efficacy of these DMTs in improving the prognosis for SMA patients, uncertainties persist regarding their cost-effectiveness. Evidence of their long-term clinical efficacy is lacking. Since the introduction of DMTs, there has been an increase in studies examining their cost-effectiveness across various locations. Several reviews have been conducted to analyse the economic evidence and provide summaries of individual economic studies. These reviews describe the diverse modelling approaches used to evaluate cost-effectiveness and offer guidance for future economic analyses [10, 25–27]. However, our assessment of these reviews using the assessing of multiple systematic reviews (AMSTAR) checklist [28] revealed limitations in methodology, review comprehensiveness, and inclusion/exclusion criteria. (The completed checklist is provided in Supplementary Material). Therefore, the aim of this evidence synthesis is to systematically review and critically appraise economic evaluations that assessed the cost-effectiveness of DMTs for the treatment of people living with SMA.

## Methods

A systematic literature review (SLR) of all economic evidence on the cost-effectiveness of DMTs for treating SMA was performed following the principles outlined in the Cochrane Handbook for Systematic Reviews of Interventions [29] A flow diagram illustrating the number of records identified, included and excluded studies at each stage of the SLR is presented according to the Preferred Reporting Items in Systematic Reviews and Meta-analyses (PRISMA) guidelines [30].

### Search strategy

The searches comprised:

1. Searching of electronic bibliographic databases, websites of health technology/medicines assessment agencies and internet (Google) searching
2. Scrutiny of references of studies included and a selection of recent, relevant systematic reviews.

A comprehensive search strategy was developed by an information specialist in collaboration with the review team to cover SMA treatments: onasemnogene abeparvovec, nusinersen, and risdiplam. The searches included validated filters for economic evaluations and combinations of free text keywords and indexing terms (MeSH/EMTREE) for the population and interventions. The strategy was first developed in Embase (via Ovid), reviewed by another specialist, and adapted for various databases. Searches were conducted in Embase (Ovid), MEDLINE (Ovid), International HTA database (INAHTA), Web of Science, CEA Registry, EconPapers, and supplemented by targeted Google searches and examination of selected health technology assessment and regulatory agency websites. Searches were initially conducted in January 2024, then updated on 10^th^-11^th^ February 2025. Details of the search strategy can be found in Supplementary Material. Search results were exported to EndNote 21, where duplicate records were systematically identified and removed.

### Inclusion criteria

Reviewers piloted a screening form based on predefined inclusion criteria. The selection process involved two stages: screening titles/abstracts and then an evaluation of full text articles for eligibility. Two reviewers independently screened records from searches, with potentially relevant titles/abstracts progressing to the full text stage. In the screening and eligibility phases, the inclusion criteria encompassed:

- All types of economic evaluations (cost-effectiveness, cost-utility, cost-benefit, cost-consequence, or cost-minimisation analyses).
- Any healthcare setting, with the aim of being as inclusive as possible.
- Interventions or comparators defined as best supportive care (BSC), onasemnogene aberparvovec, nusinersen or risdiplam.
- Outcomes reported in terms of an incremental cost-effectiveness ratio (ICER), expressed as cost per life-years gained (LYG) or cost per quality-adjusted life years (QALYs).
- Only full publications in the English language were considered

Full texts of agreed abstracts were independently reviewed by both reviewers, and disagreements were resolved through discussion or consultation with a third reviewer. The study flow and reasons for excluding full-text articles were documented using the PRISMA flow diagram.[30].

### Data extraction and quality appraisal

Information from relevant studies was extracted using a pre-piloted data extraction form based on Wijnen et al. [31] Details included study characteristics, treatment strategies, analytical methods, results, discussion points, and other (e.g. source of funding). For data that was missing or not clearly reported, efforts were made to contact the corresponding author. A request for the missing information was sent, allowing authors a two-week period to respond. If no response was received within the specified timeframe, we assumed that the requested information was not available. Data extraction was performed by one reviewer and accuracy was checked by a second, with disagreements resolved by discussion or involving a third reviewer. Data extraction sheets are available on request.

All economic evaluations were comprehensive appraisal. The evaluations were assessed for reporting and methodological quality using CHEERS and Philips’ checklists [32, 33] CHEERS focuses on relevance to policy, transparency, and result reporting, while the Philips’ checklist looks at methodological quality for risk of bias with 57 items in structure, data, and consistency domains. Each economic analysis was assessed by one reviewer and cross-checked by a second reviewer. Any disagreements between the reviewers were resolved by discussion or by recourse to a third reviewer. Completed quality assessment forms are reported in supplementary material.

### Synthesis of cost-effectiveness evidence

Data from the selected studies was synthesised and displayed in tabular format. Given the context-dependent nature of economic evaluations, all studies were summarised descriptively to highlight key determinants of cost-effectiveness and sources of uncertainty identified. We examined cost-effectiveness data in detail, and proposed recommendations to inform future economic modelling practices.

## Results

**Fig. 1:**
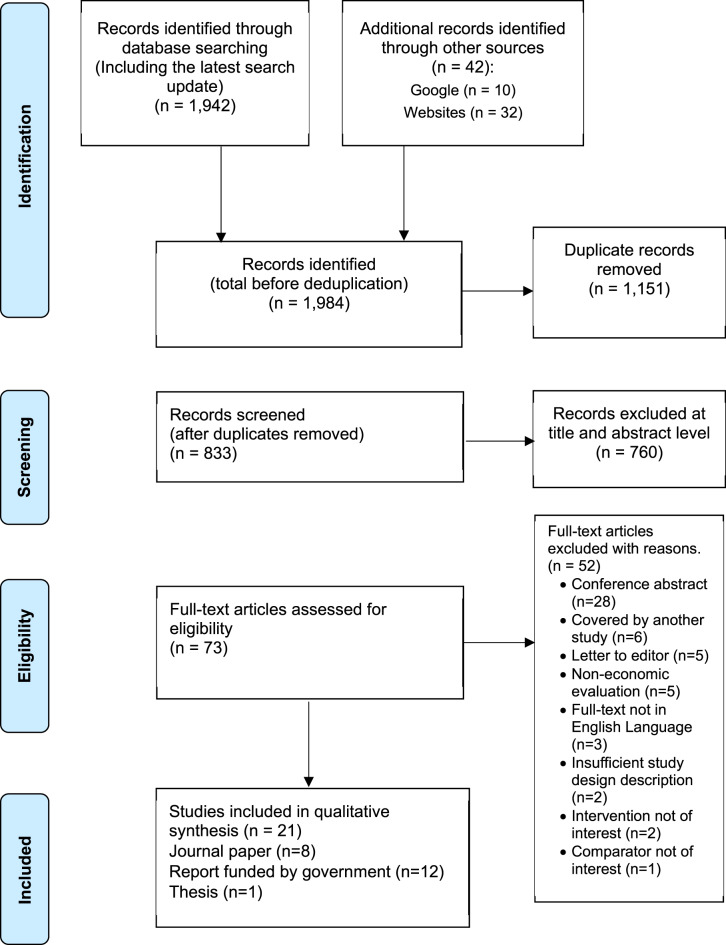
PRISMA flow diagram for studies included in the study selection process

Searching electronic databases and other sources (original and updated searches) resulted in 1,984 records. After deduplication, 833 records remained for screening. Following title and abstract assessment, 760 records were excluded, and 73 studies proceeded to full text evaluation. Eventually, 21 studies (eight journal papers [12, 34–40], 12 government-funded reports [41–52], and one doctoral thesis [53]) were eligible for inclusion and underwent data extraction, synthesis and quality appraisal. Detailed information on the selection process and reasons for exclusions can be found in Fig. 1.

### Key characteristics of included studies

#### Population

The main study characteristics are summarised in Table 1. Across the 21 included economic analyses studies, the populations of interest were people with presymptomatic SMA, and Types 1, 2 and 3 SMA. No economic analyses were conducted for Type 0 or Type 4 SMA. Subgroup categorisation based on onset age and disease duration, particularly distinguishing infantile-onset and later-onset types, showcases efforts to capture the complexity of SMA [3, 38, 42, 48, 51] Notable variability includes a focus on pre-symptomatic newborns [44], emphasis on infants with SMA Type 1 at a mean age of 3.4 months [34] and diverging aims concerning SMA Type 1 [37, 52] These differences reflect varied research objectives, designs, and patient selection contributing to defined subgroup delineations.

**Table 1:** Characteristics of the economic analyses included in the systematic review

Study, year	Target population and subgroup(s)	Intervention(s), Comparator(s) Outcome(s), Study perspective, Location & Setting	Model structure and health states	Time horizon, cycle length and discount rate	Total costs^1&3^ (intervention and comparator(s)) Source of inputs	Total QALYs^3^ (intervention and comparator(s)) Source of inputs	Base-case ICER^2^ (cost per QALY) and WTP thresholds
1) NICE-Nusinersen TA588, 2019 [41]	Target population: Paediatric patients with 5q SMA (Types 1,2 and 3) Subgroups: 1- infantile Infantile-onset (type 1) • ≤12 weeks disease duration • > 12 weeks disease duration 2- Later-onset ((types II and III) SMA.) • < 25 months disease duration • ≥25 months disease duration	Intervention: Nusinersen Comparator: BSC Outcomes: LYG, QALYs Study perspective: NHS and PSS of UK Location (Setting): UK (relevant settings (e.g. inpatient, outpatient/clinic)	Two Markov models: • Infantile onset (type 1) • Later-onset (type 2/3) Health states: Type 1: Eight health states based on HINE-2 including: No milestone achieved, mild milestones, moderate milestones, sits without support, stands with assistance, walks with assistance, stands/walks unaided and dead. Type 2/3: Seven states based on HMFSE and WHO including: Sits without support but does not roll, sits and rolls independently, sits and crawls with hands and knees, stands/walks with assistance, stands unaided, walks unaided and dead.	Time horizon: • Type 1: 60 years • Type 2/3: 80 years Cycle length: • Type 1: new cycle after 2, 6, 10, 13 and 14 months and every 4 months thereafter • Type 2/3: new cycle after 3, 6, 9, 12 and 15 months and every 4 months thereafter Discount rate: • 3.5% per year for both costs and benefits	Early onset (patient, and patient and caregiver): • Nusinersen: £2,260,700 • BSC: £71,500 Later-onset (patient, and patient and caregiver): • Nusinersen: £3,153,300 • BSC: £184,300 Sources of costs: Real-world evidence survey and data from the Hospital Episode Statistics in the UK	Early onset (patient): • Nusinersen: 7.83 • BSC: 2.49 Later-onset (patient): • Nusinersen: 16.76 • BSC: 14.52 Early onset (patient and caregiver): • Nusinersen: 7.58 • BSC: 2.17 Later-onset (patient and caregiver): • Nusinersen: 15.50 • BSC: 12.36 Source of utilities: Manufacturer experts for patients, and literature for caregivers.	Early onset (patient): Nusinersen vs BSC: £410,000 Later-onset (patients): Nusinersen vs BSC: £404,700 Early onset (patient and carer): Nusinersen vs BSC: £1,325,800 WTP thresholds: £30,000/QALY
2) NICE-Risdiplam TA755, 2021 [42]	Target population: • Paediatric patients with 5q SMA (Types 1,2 and 3) Subgroups: • Type 2/3: included both ambulant and non-ambulant patients aged 2–25 years at the time of enrolment with type 2 and 3 SMA. • Type 1: included infants with symptomatic type 1 SMA aged 1–7 months at the time of enrolment.	Intervention: Risdiplam Comparator: BSC Outcomes: LYG, QALYs Study perspective: NHS and PSS of UK Location (Setting): UK (relevant settings (e.g. inpatient, outpatient/clinic)	Two Markov models: • Type 2/3 model • Type 1 model Health states: Type 2/3: Six states based on MFM-32 and HMFSE (‘not sitting’, ‘sitting with support’, ‘sitting unsupported’, ‘standing (with or without support)’ and ‘walking (with or without support)’ and ‘death’. (Plateau at 26 months) Type 1: Six states based on HINE-2 (‘not sitting’, ‘sitting’, ‘standing’, ‘walking’) and ‘permanent ventilation’ and ‘death’. (Plateau at 66 months)	Time horizon: • Type 1 and Type 2/3: 90 years Cycle length: • Type 1 and Type 2/3: one month. Discount rate: • 3.5% per year for both costs and benefits	Commercial in confidence Sources of costs: NICE-Nusinersen TA588, NHS Reference Costs, permanent ventilation costs 175% higher than non-sitting health states.	Type 1 SMA(patient): • Risdiplam: 4.77 • BSC: 0.02 Type 1 SMA (carers): • Risdiplam: −6.68 • BSC: −3.14 Type 2/3 SMA(patient): • Risdiplam: 11.31 • BSC: 5.98 Type 2/3 SMA (carers): • Risdiplam: −3.71 • BSC: −10.06 Source of utilities: NICE-Nusinersen TA588 for patients, and ERGs methodology linking caregiver distress to the patient’s health state for caregivers.	Commercial in confidence Risdiplam’s cost-effectiveness exceeded NICEs acceptable NHS threshold for routine use. WTP thresholds: £30,000/QALY
3) NICE-OA HST15-type1, 2021 [43]	patients with 5q SMA- Infantile-onset (type 1)	Intervention: OA Comparator: BSC Outcomes: LYG, QALYs Study perspective: NHS and PSS of UK Location (Setting): UK (relevant settings (e.g. inpatient, outpatient/clinic)	One Markov model Health states: Six states: not sitting, permanent assisted ventilation, sits unassisted, walks unassisted, within a broad range of normal development and dead.	Time horizon: 100 years Cycle length: 6-month cycles for first 3 years, 12-month cycles for remainder of model Discount rate: • 3.5% per year for both costs and benefits	OA: £2,640,022 BSC: £381,131 Sources of costs: Inpatient and outpatient costs: NHS pharmacological therapy resources: PCA	OA: 10.007 BSC: 0.210 Source of utilities: PAV: Assumption based on the ERG interim report, Not sitting – BSC and OA: Thompson et al. 2017 [54], Sits unassisted – BSC and OA: Tappenden et al. 2018 [55], Walks unassisted and Broad range of normal development: Ara and Brazier 2010 [56]	OA vs BSC: $230,568 WTP thresholds: £30,000/QALY
4) NICE-OA HST24-pre-symptomatic, 2023 [44]	Newborn infants with genetically confirmed, pre-symptomatic SMA with two or three copies of the *SMN2* gene who were age ≤6 weeks (≤42 days) at time of treatment.	Intervention: OA Comparator: BSC Outcomes: LYG, QALYs Study perspective: NHS and PSS of UK Location (Setting): UK (relevant settings (e.g. inpatient, outpatient/clinic)	Two Markov model: • Short-term model • Long-term extrapolation model Health states: Short-term: Six states including: Sitting and walking independently, non-sitter and no PAV, non-sitter with PAV, sitter, delayed walker, death Long-term: Seven states including six states from short term and experiences later onset SMA	Time horizon: 100 years Cycle length: One month Discount rate: • 3.5% per year for both costs and benefits	OA: 2,096,927 BSC: 882,564 Sources of costs: Costs sourced from NHS, the NHS Business Services Authority prescription cost analysis, and the literature.	Total QALYs are in commercial in confidence Incremental QALYs: 17.20 Source of utilities: PAV: Assumption based on the ERG interim report. Not sitting and Patients in the walking & experiencing later onset SMA state who lase the ability to walk: Thompson et al. 2017 [54] Sitting: EAGs clinical experts in TA588. Walking and broad range of normal development: Ara and Brazier 2010 [56]	OA vs BSC: 70,610 WTP thresholds: £30,000/QALY
5) ICER- Nusinersen & OA, 2019 [45]	SMA patients of all ages and types including: • symptomatic patients with infantile-onset (Type I) SMA • symptomatic patients with later-onset (Type II/III) SMA • presymptomatic SMA patients	Intervention: Nusinersen and OA Comparator: BSC Outcomes: LYG, QALYs Study perspective: USA health care sector Location (Setting): USA (inpatient, outpatient/clinic, office, and home settings)	Three de novo Markov models in accordance with target population. Health states: • Type I and Pre-symptomatic models: ‘Not sitting’, ‘PV’, ‘sitting’, ‘walking’, ‘death’ • Type II/III: ‘sitting’, ‘walking’, ‘death’ Absorbing states For long-term extrapolation model: Type I: ‘PV’ and ‘death’ Type II/III: ‘death’	Time horizon: Lifetime Cycle length: One month Discount rate: 3.0% for costs and benefits	Infantile-onset: • Nusinersen: $3,884,000 • BSC: $789,000 Infantile-onset: • OA: $3,657,000 • BSC: $789,000 Later-onset: • Nusinersen:$9,148,000 • BSC: $1,442,000 Pre-symptomatic: • Nusinersen: $11,929,000 • BSC: $801,000 Sources of costs: Inpatient Cost: Nationwide Children’s Hospital, Physician fee schedule 2018 [57]; facility price Redbook 2018 for administration costs [58]	Infantile-onset: • Nusinersen: 3.24 • BSC: 0.46 Infantile-onset: • OA: 12.23 • BSC: 0.46 Later-onset: • Nusinersen: 12.28 • BSC: 11.34 Pre-symptomatic: • Nusinersen: 21.94 • BSC: 6.25 Source of utilities: PAV for both arm, Not sitting for BSC arm: Thompson et al. 2017 [54] Not sitting for treatment arm: Assumption Sitting for BSC arm: Tappenden et al. 2018 [55] Sitting for treatment arm: Assumption Walking for both arms: General population utility	Infantile onset: • Nusinersen vs BSC: $1,112,000 Infantile onset: • OA vs BSC: $243,000 Later-onset: • Nusinersen vs BSC: $8,156,000 Pre- symptomatic: • Nusinersen vs BSC: $709,000 WTP thresholds: $150,000/QALY
6) CADTH- Nusinersen, 2019 [46]	Patients with SMA (type 1, 2 and 3)	Intervention: Nusinersen Comparator: RWC Outcomes: LYG, QALYs Study perspective: Canadian public health care system Location (Setting): Canada	Three Markov models: type I, type II, and type III Health states: SMA Type I (10 health states): Baseline, improved baseline, worsened baseline, no improvement, sits without support, stands with assistance, walks with assistance, stand/walks unaided, loss of type II/III motor function, death. SMA Type II (10 health states): Baseline, worsened, no/mild/moderate improvement, stand/walk with assistance, stand unaided, walks unaided, loss of ambulation (with/without assistance), death. SMA Type III (4 health states): Baseline, non-ambulatory, ambulatory, death	Time horizon: • type 1: 25 years • type 2: 50 years • type 3: 80 years Cycle length: • type 1: At 2, 6, 10, 13, and 14 months. Subsequent cycles were every four months. • type 2: three months (For the first 15 months). Subsequent cycles were every four months. • type 3: three months (for the first 27 months), subsequent cycles every 4 months. Discount rate: 1.5% costs and benefits per annum	SMA type I: • Nusinersen: $3,534,854 • RWC: $339,683 SMA type II: • Nusinersen: $8,336.271 • RWC: $708,620 SMA type III: • Nusinersen: $5,554,707 • RWC: $1,091,307 Sources of costs: Health costs appear to be derived from a study of SMA costs in Germany (Klug et al. study) [59]	SMA type I: • Nusinersen: 3.919 • RWC: −0.881 SMA type II: • Nusinersen: 23.278 • RWC: 19.602 SMA type III: • Nusinersen: 12.053 • RWC: 10.490 Source of utilities: Patient: Types 1 and 3 SMA: unpublished utility value analyses by five experts in SMA Type 2 SMA: QoL data from CHERISH mapped to EQ-5D	• SMA type I: Nusinersen vs RWC: $665,570 • SMA type II: Nusinersen vs RWC: $2,075,435 • SMA type III: Nusinersen vs RWC: $2,855,818 WTP thresholds: Lower than $50,000/QALY
7) CADTH-Risdiplam, 2021 [47]	Patients with SMA (type 1, 2 and 3)	Intervention: Risdiplam Comparator: BSC Outcomes: QALYs Study perspective: Canadian publicly funded health care payer Location (Setting): Canada	Two Markov models in accordance with target population. Health states: • SMA Type I (6 health states): Not sitting, sitting, standing, walking, permanent ventilation, death. • SMA Type II/III (6 health states): Not sitting, supported sitting, unsupported sitting, standing, walking, death.	Time horizon: • Type I: 25 years • Type II/III: 80 years Cycle length: For both models one month Discount rate: 1.5% costs and benefits per annum.	Type I: • BSC: 68,293 • Risdiplam: 961,580 • Nusinersen: 1,865,665 Type II/III: • BSC: 825,656 • Risdiplam: 11,454,583 • Nusinersen: 11,892,866 Sources of costs: Canadian burden-of-illness study and alberta Hospital [60], the Ontario Ministry of Health [61, 62] End-of-life costs were obtained from Widger et al. [63] and Seow et al.[64]	Type I: • BSC: 3.53 • Risdiplam: 10.19 • Nusinersen: 10.17 Type II/III: • BSC: 70.11 • Risdiplam: 71.04 • Nusinersen: 70.91 Source of utilities: Sponsor-commissioned Canadian burden-of-illness survey. “Supported sitting” utility applied to “permanent ventilation” and “not sitting” states.	Type I: Risdiplam vs BSC: 134,229 Risdiplam vs Nusinersen: Dominant Type II/III: Risdiplam vs BSC: 1,203,108 Risdiplam vs Nusinersen: Dominant WTP thresholds: Lower than $50,000/QALY
8) CADTH-OA, 2021 [48]	Patients with SMA type 1 with an onset of symptoms at ≤ 6 months of age, and with 2 copies of the SMN2 gene.	Intervention: OA Comparator: BSC and Nusinersen Outcomes: QALYs Study perspective: Canadian publicly funded health care payer Location (Setting): Canada	One Markov model Health states: Six Health States: Within a broad range of normal development, walking unassisted, sitting unassisted, unable to sit unassisted, PAV, death.	Time horizon: 60 years Cycle length: Six 6 months in the early phase and yearly in the extrapolation phase. Discount rate: 1.5% costs and benefits per annum.	BSC: 132,600 OA: 3,266,544 Nusinersen: 3,938,147 Sources of costs: • Administration costs: Ontario Schedule of Benefits for physician services. • Screening costs: expert opinion. • Health state costs: unpublished health care resource utilization study conducted by the sponsor.	BSC: 0.21 OA: 10.89 Nusinersen: 4.54 Source of utilities: Utilities for unable to sit and sitting unassisted from Tappenden et al. (2018) [55]; general population utilities for “walking unassisted” and “within normal development” from Ara et al., 2010) [56]	OA vs BSC: 293,521 OA vs BSC: Dominant Nusinersen vs BSC: Dominated WTP thresholds: Lower than $50,000/QALY
9) Meijer et al., 2023 [53]	Patients with SMA Type I, SMN1 mutation, 2 SMN2 copies, treated before 6 months	Intervention: OA Comparator: BSC Outcomes: QALY Study perspective: Societal perspective Location (Setting): Netherlands	One Markov model Health states: Five health states: Not sitting and PAV-free, PAV, sitting independently, walking independently, dead.	Time horizon: 36 months for short-term and 99 years for long-term Cycle length: monthly Discount rate: 4% for costs and 1.5% for benefits	Type I: • BSC: €350,878 • OA: €4,386,381 pre-symptomatic • BSC: €350,878 • OA: €3,951,500 Sources of costs: Literature (study conducted by Klug et al. in Germany in 2016) [59]	Type I: • BSC: 0.37 • OA: 16.03 pre-symptomatic • BSC: 0.37 • OA: 28.70 Source of inputs: Literatures (mainly ZIN report of nusinersen and the comments provided in the ZIN report of OA) [65, 66]	Type I: • OA vs BSC: € 257,717 pre-symptomatic • OA vs BSC: € 127,107 WTP thresholds: €80,000/QALY
10) Broekhoff et al., 2021 [34]	Infants born with SMA I- Mean age (months) = 3.4	Intervention: OA Comparator: BSC and Nusinersen Outcomes: QALYs Study perspective: societal perspective Location (Setting): Netherlands	One Markov model Health states: five health states: three states corresponding to SMA types (SMA I-III), one for PV, and one for death.	Time horizon: 100 years Cycle length: one month Discount rate: 4% for Costs and 1.5% for benefits	BSC: 922,130 OA: 4,024,879 Nusinersen: 3,002,379 Sources of costs: published literature (the ZIN report, which uses cost calculations by Klug et al.) [59]	BSC: 4.415 OA: 26.757 Nusinersen: 7.625 Sources of utilities: Dutch National Health Care Institute (ZIN) model	OA vs BSC: 138,875 Nusinersen vs BSC: 647,850 OA vs Nusinersen: 53,477 WTP thresholds: €80,000/QALY
11) SMC nusinersen, 2018 [49]	patients with symptomatic type 1 SMA	Intervention: Nusinersen with BSC Comparator: BSC Outcomes: QALY, YLG Study perspective: NHS Scotland and social work Location (Setting): Scotland	Two Markov models (type I SMA and type II or III SMA) Health states: A 10-state model includes health progression through worsening, stabilizing, and improving states, as well as functional milestones such as “sitting without support” and “standing without assistance.” The model for later-onset SMA patients mirrors the infant model but incorporates state adjustments tailored to Type II and Type III SMA.	Time horizon: 80 years Cycle length: Unclear Discount rate: Unclear	Infantile model: • incremental cost: £2,151,509 Later-onset model: • incremental cost: £3,728,246 Sources of costs: Unclear	Infantile model: • Incremental QALYs: 5.02 Later-onset model: • Incremental QALYs: 2.29 Sources of utilities: Later-onset health utilities were derived from CHERISH PedsQL data converted to EQ-5D, while infant values were adjusted for infant-specific characteristics.	Infantile model: £428,964 Infantile model: £1,624,951 WTP thresholds: £30,000/QALY
12) SMC -OA, 2021 [50]	Patients with SMA with a bi-allelic mutation in the SMN1 gene (or up to 3 copies of the SMN2 gene) and a clinical diagnosis of SMA type 1.	Intervention: OA Comparator: nusinersen Outcomes: QALY, LYG Study perspective: health and social care Location (Setting): Scotland	One Markov models Health states: The model includes six states: normal development, unassisted walking, unassisted sitting, inability to sit, reliance on assisted ventilation, and death.	Time horizon: lifetime Cycle length: Six months for the first three years and annually thereafter Discount rate: Unclear	OA: £2,704,737 Nusinersen: £2,800,590 Sources of costs: Routine unit cost sources were applied to estimate costs, and this was tested in scenario analysis using Scottish Health Service Costs where available.	OA: 7.50 Nusinersen: 3.77 Sources of utilities: Utility values were sourced from the literature using proxy parental or carer values and/or clinical advice.	OA vs Nusinersen (£/QALY): −25,740 WTP thresholds: £30,000/QALY
13) SMC risdiplam, 2022 [51]	Infants with symptomatic type 1 SMA aged 2–7 months were used in the type 1 model, while the type 2/3 model included ambulant and non-ambulant patients aged 2–25 years.	Intervention: risdiplam with BSC Comparator: nusinersen with BSC Outcomes: QALY Study perspective: Unclear (maybe payer and social perspective) Setting: Unclear Location: Scotland	Two Markov models: (type I SMA and type II or III SMA) Health states: Common motor milestone states (in both models): not sitting, sitting, standing, and walking. The Type 2/3 model split sitting into with/without support, while the Type 1 model included permanent ventilation. Death was absorbing.	Time horizon: lifetime Cycle length: one month Discount rate: Unclear	Not reported Sources of costs: Costs for drug acquisition, administration, and resource use of risdiplam and nusinersen were factored in from real-world studies by their respective companies.	Not reported Sources of utilities: literature sources or via a previous NICE submission TA588	Risdiplam is estimated to be more effective and less costly than nusinersen at list prices. WTP thresholds: £30,000/QALY
14) Connock et al., 2020 [12]	Patients with SMA Type 1	Intervention: OA Comparator: BSC and nusinersen Outcomes: QALY Study perspective: UK NHS perspective Location (Setting): UK	One Markov model Health states: The authors mentioned: “We identified several Biogen-sponsored cost and cost-utility studies comparing nusinersen versus BSC using the same Markov model structure; one of these adopted a UK NHS perspective (NICE-Nusinersen TA588, 2019) and was included.”	Time horizon: Lifetime Cycle length: Unclear Discount rate: 3.5% for both costs and benefits	Nusinersen: • 4,352,213 (From source A (Malone et al.) [36] • 2,258,852 (From source B (NICE- TA588) [67] OA: • 2,903,706 (From source A (Malone et al.) (for a 2.5 USD million acquisition price) • 4,576,047 (From source B (Malone et al.) (for a 5 USD million acquisition price) BSC: • 26,637 (From Biogen submission to NICE)	Nusinersen: • 5.29 (From Malone et al.) • 7.86 (From NICE TA588) OA: • 15.65 (From Malone et al. [36] (for a 2.5 USD million acquisition price) • 15.65 (From Malone et al. (for a 5 USD million acquisition price) BSC: • 2.58 (From NICE TA588)	ICER vs BSC: Nusinersen in all models is dominated. OA drug cost US$5 m: OA (Malone et al. data): £343, 209 OA (Biogen data): (£343,209) OA drug cost US$2.5 m: • OA (Malone et al. data): £215,257 • OA: (Biogen data: £215,257 WTP thresholds: £30,000/QALY
15) Dean et al., 2021 [35]	Symptomatic SMA type 1 patients	Intervention: OA Comparator: Nusinersen, BSC Outcomes: QALY Study perspective: Commercial payer OF USA Location (Setting): USA	One Markov model Health states: The states of the model are like the “ICER - Nusinersen & OA 2019” study Markov model for Type I SMA.[45]	Time horizon: Lifetime Cycle length: 6-monthly for first 3 years then annually Discount rate: 3% for both costs and benefits	BSC: $1,961,710 Nusinersen: $4,602,692 Oa: $3,930,879 Sources of costs: Price of OA $2.125 M one-time dose, other costs were equal to costs used in the ICER analysis (2019) [45], cost of ventilator estimated from a UK study(Noyes et al. 2006) [68]	BSC: 1.15 Nusinersen: 2.88 OA: 13.33 Sources of utilities: Equivalent to weightings used in the ICER analysis [45]	OA vs BSC: $161,648 OA vs Nusinersen: dominant WTP thresholds: $150,000/QALY
16) Malone et al., 2019 [36]	Paediatric with genetically confirmed SMA1 and two copies of SMN2, diagnosed before six months of age, and receiving either the recommended therapeutic amount of OA or nusinersen, along with BSC	Intervention: OA Comparator: Nusinersen Outcomes: QALY, LYG Study perspective: Commercial insurer in the U.S. Location (Setting): USA	One Markov model Health States: Six health states, including “Within a broad range of normal development,” “Walking functionally equivalent to SMA Type III,” “Sitting functionally equivalent to SMA Type II,” “Not sitting and living ventilation-free,” “Not sitting and requiring permanent assisted ventilation,” and “Death.”	Time horizon: 100 years Cycle length: Six months for the first three years, and then 12 months for all cycles thereafter Discount rate: 3% for both costs and benefits	• OA $2.5 M: $4,214,379 • OA $3 M: $4,699,816 • OA $4 M: $5,670,690 • OA $5 M: $6,641,564 Nusinersen: $6,316,711 Sources of costs: SMA patient costs in USA: commercial plans by age at first claim [69] Nusinersen product cost: based on manufacturer [70] Nusinersen administration cost: Micro-costed. Hospital stays for anesthesia-related complications: Graham et al.[71]	• OA: 15.65 • Nusinersen: 5.29 Sources of utilities: The model incorporated utility values derived from the CHERISH (PedsQL data) clinical trial of nusinersen for later-onset SMA (NCT02292537), which were transformed to the EQ-5D youth version through a documented algorithm.[54]	• OA $2.5 M vs Nusinersen: Dominant • OA $3 M vs Nusinersen: Dominant • OA $4 M vs Nusinersen: Dominant • OA $5 M vs Nusinersen: $31,379 WTP thresholds: $150,000/QALY
17) Wang et al., 2022 [37]	Infants born with SMA Type I as the recruited patients in the clinical trials of nusinersen and OA	Intervention: OA Comparator: SOC and nusinersen Outcomes: QALY Study perspective: Australian healthcare system Location (Setting): Australian	One Markov model Health states: Five health states, including: “not sitting and PAV-free,” “sitting independently,” “walking independently,” and “dead.”	Time horizon: 100 years Cycle length: Monthly Discount rate: 5% annually for both costs and benefits	SOC: $923,335 Nusinersen: $2,592,526 OA: $5,034,806 Sources of costs: Drug acquisition and administration costs from: PBS [72], MBS [73], NHCDC [74]. Health state costs: Recently published study in Australia [75] AND CER Institute [45]	SOC: 0.301 Nusinersen: 0.602 OA: 2.574 Sources of utilities: Utility values come from Chambers et al.2020 [75]	• Nusinersen vs SOC: $2,772,798 • OA vs SOC: $1,808,471 • OA vs nusinersen: $1,238,288 WTP thresholds: $50,000/QALY
18) Zuluaga-Sanchez et al., 2019 [38]	Patients with infantile-onset and later-onset SMA	Intervention: Nusinersen and SoC Comparator: SoC Outcomes: QALY Study perspective: societal Location (Setting): Sweden	Two Markov model Health states: Infantile-onset: Ten states including: baseline, worsened, stabilisation of baseline, improved, stands/walks unaided, sits unaided, stands aided, walks aided, loss of later-onset motor function, death. Later-Onset: Ten states including baseline, worsened, stabilised, mildly improved, moderately improved, stands unaided, walks unaided, stands/walks with assistance, loss of ambulation with/without assistance, death.	Time horizon: 40 years for infantile-onset and 80 years for later-onset Cycle length: In trial follow-ups for ENDEAR: days 1, 64, 183, 302 and 394; CHERISH: days 1, 92, 169, 274, 365 and 456; after follow-ups: four months Discount rate: 3.0% for both costs and benefits	Infantile model: • Nusinersen + SoC: 22,970,891 (SEK) • SoC: 1,513,607 (SEK) Later-onset model: • Nusinersen + SoC: 64,095,327 (SEK) • SoC: 25,175,193 (SEK) Sources of costs: German study (Klug et al. 2016) [59] and a Spanish study (López-Bastida et al. 2017) [76] Country-specific unit costs were mainly collected from hospital price lists.	Infantile model: • Nusinersen + SoC: 3.65 • SoC: −0.20 Later-onset model: • Nusinersen + SoC: 9.25 • SoC: − 0.29 Sources of utilities: Health-state utility values come from Lloyd et al. 2017 [77] The EQ-5D (youth version) was scored using the EQ-5D-3 L UK preference weights.	Infantile-onset: Nusinersen + SoC vs SoC = 5,562,027 (SEK) Later-onset: Nusinersen + SoC vs SoC = 4,079,635 (SEK) WTP thresholds: 2 million SEK (€195,600)/QALY
19) Thokala et al., 2020 [39]	Patients with infantile-onset SMA (type 1)	Intervention: Nusinersen Comparator: BSC Outcomes: LYG, QALY Study perspective: US health care sector Location (Setting): US	One Markov model The model contained two parts: (1) a short term phase concordant with clinical study data, and (2) a long-term extrapolation model. Health states: Five main health states, including: permanent ventilation, not sitting, sitting, walking, and death.	Time horizon: lifetime Cycle length: monthly Discount rate: 3% per annum for both costs and benefits	Nusinersen: $3,884,000 BSC: $789,000 Sources of costs: Nusinersen treatment cost: Magellan 2016 [78], Redbook 2018.[58] Administration cost: Physician fee schedule 2018; Nationwide Children’s Hospital [57] The health care utilization costs: Shieh et al. 2017 [69]	Nusinersen: 3.24 BSC: 0.46 Sources of utilities: Permanent ventilation from Thompson et al., 2017.[54] Not sitting in BSC arm Same as “permanent ventilation” and for nusinersen based on assumption Sitting for BSC based on Tappenden et al., 2018 [55] and for nusinersen based on assumption	Nusinersen vs BSC: $1,112,000 WTP thresholds: $150,000/QALY
20) NCPE-2017 [52]	infantile and later onset SMA	Intervention: Nusinersen Comparator: SOC Outcomes: QALY, LYG Study perspective: Health Service Executive Location (Setting): Ireland	Two Markov models (infantile and later onset SMA) Health states: Unclear	Time horizon: Lifetime Cycle length: determined by motor assessments timing and maintenance dose administration. Discount rate: 5% for costs and benefits	Not reported Sources of costs: Unclear	Not reported Sources of utilities: PedsQL data from CHERISH trial determined later-onset SMA utilities, impacting infantile SMA model. Data mapped to EQ5D scale for utility derivation.	infantile onset SMA: Nusinersen vs SOC: €501,069 later onset SMA: €2,163,798 WTP thresholds: €45,000/QALY
21) Khuntha S, et al., 2025 [40]	Symptomatic SMA patients categorized into two groups: SMA type 1 age onset at 3 months, SMA type 2 age onset at 12 years, and SMA type 3 age onset at 18 months	Intervention: onasemnogene, risdiplam Comparator: SOC Outcomes: LYG, QALY Study perspective: Societal perspective Location (Setting): Thailand	Two same Markov models for type 1 and types 2/3 Health states: Six health states including independentlly walking, walking with support, Siting without support/standing, not-sitting and not requiring PAV, PAV, and death	Time horizon: lifetime Cycle length: 6-month Discount rate: 3% per annum for both costs and benefits	SMA type 1 (USD): Onasemnogene • Provider: 1,342,000 • Societal: 1,900,000 Risdiplam • Provider: 942,000 • Societal: 1,045,000 SOC • Provider: 53,000 • Societal: 294,000 SMA type 2–3: Risdiplam • Provider: 2,569,000 • Societal: 2,753,000 SOC • Provider: 82,000 • Societal: 211,000 Sources of costs: Medical Costs: Retrieved from hospital records (2016–2021) and adjusted using the charge-to-cost ratio. New Treatment Costs: Provided by pharmaceutical companies. Non-Medical & Indirect Costs: Collected via patient and caregiver interviews (June 2022–Feb 2023).	SMA type 1 Onasemnogene • Provider: 11.66 • Societal: 11.66 Risdiplam • Provider: 6.56 • Societal: 6.56 SOC • Provider: 1.82 • Societal: 1.82 SMA type 2–3: Risdiplam • Provider: 15.83 • Societal: 15.83 SOC • Provider: 10.71 • Societal: 10.71 Sources of utilities: EQ-5D-Y (Thai version): Used for patient and caregiver interviews. Utility Values: Derived from Japan’s EQ-5D-Y tariff due to the absence of a Thai valuation study. Additional Estimates: Other health state values were extrapolated using percentage differences from a Chinese study on SMA patients.	SMA type 1 (USD/QALY gained) Onasemnogene • Provider: 130,908 • Societal: 163,102 Risdiplam • Provider: 187,456 • Societal: 158,357 SMA type 2–3: Risdiplam • Provider: 485,957 • Societal: 496,704 WTP thresholds: 4,444(USD)/QALY

BSC, best supportive care; LYG, life-year gained; PSS, personal social service; QALY, quality adjusted life-year; RWE, real-world evidence; SMA, spinal muscular atrophy; OA, Onasemnogene abeparvovec; PCA, pharmacological therapy resources; permanent assisted ventilation, PAV; purchasing power parity, PPP; United States, US; Standard Of Care, SOC; Pharmaceutical Benefits Scheme, PBS; Medicare Benefits Schedule, MBS; National Hospital Cost Data Collection, NHCDC

^1^List prices have been used for acquisition drug costs

^2^We acknowledge that the ICER reported in NICE and other HTA body reports may not be final due to PAS agreements

^3^Cost and QALY values are reported as ‘total’ costs/QALYs. If a specific study does not provide total values, the type of value, such as incremental costs or QALYs, is specified

#### Health technologies compared, perspective, country

Treatments compared included onasemnogene abeparvovec, nusinersen, and risdiplam, versus BSC, onasemnogene abeparvovec versus nusinersen [12, 34–37, 50], or risdiplam versus nusinersen [51], with life-years gained (LYG) and quality adjusted life years (QALYs) as primary outcomes. Most studies adopted either a health system [12, 37, 39, 41–52] or societal perspective [34, 38, 40, 53] and were conducted in multiple countries, including the UK [12, 41–44, 49–52], USA [35, 36, 39, 45], Canada [46–48], Netherlands [34, 53], Australia [37], Sweden [38] and Thailand.[40]

#### Model structure, time horizon and discount rate

All studies employed Markov models to evaluate SMA progression and treatment cost-effectiveness, defining health states based on functional milestones such as sitting, standing, walking, and ventilation support. Variability existed in model structures, with some studies employing single, two, or three Markov models depending on SMA subtypes and analysis scope. Long-term horizons ranged from 60 to 100 years, ensuring comprehensive cost-benefit assessments. Annual discount rates of 1.5% [46–48], 3% [35, 36, 38–40, 45], 3.5% [12, 41–44, 49–51], or 5% [37, 52] were applied, reflecting different approaches to adjusting future costs and benefits. Cycle lengths varied from monthly to longer intervals, with some studies [49–51] lacking clarity on cycle lengths and discount rates.

#### Outcomes related characteristics

Health-state utility values in included studies were derived from clinical trials, literature, expert opinions, and caregiver disutility estimates. NICE technology appraisals TA588 (2019) and TA755 (2021) adjusted utility values based on caregiver disutilities for different SMA types both acknowledging caregiving impacts on utility estimates [41, 42] NICE TA588 (2019), NICE HST24 (2023) and CADTH (2019) derived patient utility values from expert analyses and trial data in a multi-model SMA context, illustrating similarities in relying on expert opinions and clinical findings.[41, 44, 46].

Differences in cost breakdown granularity existed, with some studies providing detailed itemised costs while others presented aggregated estimates. CADTH (2021) and Wang et al. (2022) both utilised national health services data for cost assessments, and exhibit similarities in leveraging local healthcare system information for cost estimations [37, 48] Comparing specific cost components from these studies could reveal insights into the consistency of cost structures within national health service contexts. SMC (2021) and Zuluaga et al. (2019), referenced specific European studies for cost data, demonstrating similarities in relying on region-specific studies for cost estimations.[38, 50]

#### Survival modelling

The primary objectives of survival extrapolation across studies were to estimate long-term overall survival (OS) and model transitions between different health states (e.g., sitting, walking, ventilation dependency) and death, supporting cost-effectiveness analyses through the calculation of life-years and QALYs, and to address the limitations inherent in rare disease datasets. Parametric models were favoured due to their ability to project beyond observed data, while non-parametric Kaplan-Meier analyses were limited primarily to untreated cohorts, such as in Meijer et al. (2023).[53]

Survival analysis in the included studies frequently drew upon two key data sources: clinical trial data and natural history data.

- Clinical trial data were utilised extensively across multiple studies to inform survival outcomes in treated cohorts [36–45, 53] These studies used a range of parametric models (e.g. Weibull and exponential) to fit to the individual patient data (IPD) or digitised KM data and extrapolate beyond the trials’ duration. The results show that Weibull, exponential, and Gompertz functional forms were common for modelling Type 1 SMA, whilst Type 2/3 SMA saw more use of spline-based models.
- Natural history data, capturing untreated disease progression, were also critical for modelling the BSC and for extrapolating beyond the limited duration of trials [34–39, 42–45, 53] Sources such as the National Network for Excellence in Neuroscience Clinical Trials (NeuroNext) Network [79, 80] and Paediatric Neuromuscular Clinical Research database (PNCR) [81] registries, provided non-interventional observational data for SMA Types 1 to 3. These were especially important in long-term extrapolations and comparative effectiveness analyses.

Model selection was guided by statistical goodness-of-fit criteria (using the Akaike Information Criterion (AIC) and Bayesian Information Criterion (BIC)) alongside visual inspection and clinical expert [34, 36–39, 42–45, 53] judgment to ensure plausibility.

#### Assumptions used in included studies

The studies evaluating SMA treatments, including NICE TA588 (2019) [41], SMC (2018) [49], and CADTH models [46–48], consistently assumed that treatment effects, particularly in maintaining motor milestones, persist beyond the study period. TA588 (2019) [41] made assumptions about treatment response, mortality, scoliosis surgery, utility values, and milestones, while also conducting scenario analyses on hazard and caregiver ratios [41] Zuluaga Sanchez et al. (2019) proposed that treatment effects continue indefinitely, factoring in milestones, mortality risk, and treatment outcomes [38] Similarly, Thokala et al. (2020) assumed sustained motor milestones until death, integrating short-term data on function, ventilation, and disease progression [39] Other studies, such as those by Wang et al. (2022) [37] and the Institute for Clinical and Economic Review (ICER) (2019) [45] assumed the continuation of treatment effects, including motor milestones and mortality risks, across various durations and patient groups. Assumptions about caregiver disutilities, treatment costs, and complications were considered in NICE TA755 (2021) [42], while modelling in CADTH (2021) [47] and Broekhoff et al. (2021) [34] focused on transitions, treatment discontinuation, and health state progression. These assumptions, including milestone sustainability, treatment efficacy, and disease progression, shape the clinical and economic outcomes in SMA assessments, influencing healthcare resource allocation and patient management strategies.

#### Characterising uncertainty across studies

Several common uncertainties were evident across the economic analyses. Clinical data uncertainty, as seen in studies like NICE HST15 (2021) [43], limits the reliability of cost-effectiveness estimates, while Zuluaga et al. (2019) [38] faced challenges due to limited resource use and cost data. Thokala et al. (2020) [39] highlighted uncertainties in survival, disease progression, and treatment effectiveness, particularly due to a lack of long-term follow-up. In Wang et al. (2022) [37], small sample sizes and insufficient long-term data limit assessments.

Studies including Dean et al. (2021) [35] and those produced by the SMC (2022) [51] raise concerns about incomplete follow-up and potential biases affecting ICER calculations. Reports from SMC (2018) and ICER (2019) [45] also faced challenges with assumptions and limited survival data. Other studies, such as NICE HST24 (2023) [44] and CADTH (2019) [46], contend with issues related to the age range in trials and unreliable utility values. In terms of characterising uncertainty across studies on SMA therapies, various analytical methods are employed. NICE TA588 (2019) [41] and Zuluaga Sanchez et al. (2019) [38] utilised probabilistic sensitivity analysis (PSA) and scenario analyses, while Thokala et al. (2020) [39] and Dean et al. (2021) [35] applied one-way sensitivity analysis (OWSA). Other studies, such as NICE TA755 (2021) [42], Wang et al. (2022) [37], and CADTH analyses [46–48], incorporated PSA, deterministic sensitivity analysis (DSA), and scenario analyses to assess cost-effectiveness.

#### Reporting and methodological quality assessment

In general, studies exhibited satisfactory reporting quality against best practice guidance on reporting of economic evaluations. While published papers typically included structured abstracts, reports often lacked them, likely due to differing requirements.

As expected, reports and monographs produced by policy bodies (NICE/SMC) provided more detailed economic analyses than published papers, though certain reports still lacked comprehensive methodological details [49–52] Approximately three-quarters of the studies reported methodological details in their economic analyses, including study population, comparators, and discount rates. However, none developed a Health Economics Analysis Plan (HEAP) or considered distributional effects. The HEAP requirements align with recent CHEERS I updates [82] The SMC reports lacked details on model selection, discount rates, and statistical analyses [49–51] With regards to the results, it was noted that six studies [12, 35, 46, 49–51] have not adequately discussed study parameters and the effect of uncertainty.

Full details on the reporting quality of each economic analysis can be found in the Supplementary Material.

Regarding methodological quality assessment, several studies, including NICE TA588 (2019) and NICE HST24 (2023) [41, 44], and ICER (2019) [45], demonstrated a high rating in terms of the best overall quality in methodology and thus, had a low risk of bias. Conversely, SMC (2021) [50] and NCPE (2017) [52] showed weaker methodological quality due to unclear decision problem definition, non-transparent structural assumptions, and inadequate handling of uncertainties. Data identification lacked transparency, and transition probabilities were poorly justified, limiting robustness for SMA treatment evaluations.

When appraising the structure, data, and consistency, Zuluaga et al. (2019) [38] and Malone et al. (2019) [36] excelled in structural aspects, ensuring clear objectives and model structure. TA588 [41] and HST24 [44] were highlighted for strong data quality, with robust parameter justification. In terms of consistency, HST24 [44] and Meijer et al. (2023) [53] were rated highly for methodological robustness.

In summary, all studies addressed methodological uncertainties to some extent and 12 considered structural uncertainties. Inconsistencies were noted in the handling of alternative assumptions regarding treatment effects and in the incorporation of uncertainty through probabilistic distributions. Thirteen of the 21 studies conducted thorough comparisons with previous models and provided explanations for any counterintuitive findings. Notably, the NICE technology appraisal reports [41–44] and the 2019 ICER [45] assessment offered more comprehensive evaluations of both internal and external validity of the results.

## Discussion

Following comprehensive searches and screening, 21 studies examining SMA were eligible for inclusion in the systematic review. Included studies highlighted a wide array of factors concerning the target population, SMA treatments, outcomes, models, time horizon, utility values, costs, analyses and limitations. Studies predominantly focused on SMA patients of Types 1 (*n* = 20), 2 (*n* = 9), and 3 (*n* = 9). No studies were undertaken with SMA Type 0 or Type 4. Markov models captured SMA progression by defining health states covering functional milestones and ventilation support and compared nusinersen, risdiplam and onasemnogene abeparvovec against BSC across different locations and settings. Time horizons ranged from 60 to 100 years, discount rates from 1.5% to 5%, and utility value sourcing highlighted varied perspectives on SMA modelling. Resource utilisation and cost implications were explored, outlining essential costs for managing SMA such as drug acquisition, administration, hospitalization, ventilation support, and outpatient care. Cost-effectiveness results were reported as incremental ratios expressed as cost per life-year and cost per QALY.

### Agreement on Markov models and health states

The selection of health states in studies related to SMA reveals notable variations in model structures, assumptions, and rationales. These differences encompass aspects such as disease progression, treatment efficacy, and mortality. Most studies employ distinct model structures tailored to specific SMA types. For example, NICE HST24 [44] utilised a two-part Markov model, distinguishing between short-term and long-term outcomes, while ICER [45] employed a single model divided into short-term and long-term phases. Both studies included presymptomatic SMA patients in their assessments, acknowledging that people may progress to Type 1 or Type 2/3 SMA over time [83] This approach highlights the importance of incorporating both short and long-term perspectives in modelling, by capturing different features of disease progression and treatment effects. A common assumption across studies is that treatment effects, particularly in maintaining motor milestones, persist beyond the observed study period. This assumption indirectly underscores the distinction between short and long-term outcomes in SMA modelling. For example, Thokala et al. (2020) assumed that patients maintain motor function milestones until death, with treatment effects continuing post-trial.[39]

The identification of health states based on motor function milestones (e.g., non-sitting, sitting, standing, and walking) appears to be a rational approach [84] However, challenges remain in defining these states, particularly when distinguishing between abilities such as sitting, standing, or walking with or without support. For example, it is necessary to determine whether these abilities should be classified as distinct health states or combined into a single state, as some prior studies have indicated that the associated costs and utilities may vary depending on the classification approach [85, 86] Resolving these issues requires more detailed data on resource use and utility changes across different health states, such as standing with or without support, which could be obtained through real-world evidence (RWE) from patient registries, observational studies, or patient-reported outcome measures. Incorporation of alternative data sources such as RWE allows for less reliance on expert opinion for utility values and resource use. This gap highlights the need for further research to refine health state definitions and improve the accuracy of Markov models in SMA.

### Long‑term modelling implications

Examining economic evaluations from various studies highlights several key limitations when evaluating the long-term impact of SMA treatments. The identified limitations stress the necessity for robust long-term efficacy and safety data to fully capture the benefits of SMA treatments over an extended period. Challenges related to treatment sustainability, including motor function milestones and QALYs, are evident across the NICE, ICER and CADTH reports. These challenges highlight uncertainty in projecting treatment outcomes over a lifetime due to the lack of long-term data, assumptions around sustained milestones, and varying patient characteristics.[27, 87]

Tailored economic evaluations are crucial to address the diverse responses within different types of SMA patient populations [41–43, 45–47, 53] Adjusting ICER per QALY values to account for these differences is crucial in accurately reflecting treatment outcomes across various patient groups with distinct disease progression rates. Efforts towards extended monitoring and real-world evidence collection are essential to bridge the gap in long-term data, enhancing the precision of evaluations and decision-making for SMA treatments.

### Source of utility values

The evaluations of utility values for patients with SMA highlights persistent challenges in deriving robust utility data for rare diseases, particularly due to limited long-term clinical data and the scarcity of validated measures for paediatric populations. Various reports produced by policy bodies such NICE, ICER, and CADTH, reveal the complexities and variations in determining utility values for different health states in SMA patients, especially in the absence of long-term clinical data. Unlike a recent systematic literature review that noted the lack of robust utility data for SMA [88], our study uniquely integrates perspectives from multiple HTA agencies and policy bodies, identifying specific methodological inconsistencies in utility estimation approaches and proposing actionable recommendations for standardisation.

A concern arises from the reliance on utility values obtained from manufacturer clinical experts, as noted in NICE TA588 (2019) [41] and TA755 (2021) [42], this risks inflating benefits and contravening established national guidelines (such as those produced by NICE [89]), which prioritises patient-derived data for utility values. To address these limitations, it would be preferable to incorporate utility values derived from patients and caregivers, supported by long-term clinical trials, observational studies, and post-marketing surveillance (i.e., RWE), as highlighted by ICER (2019) [45] Such approaches [86, 88] may help standardise utility derivation and improve transparency in the evaluation process. Multi-criteria decision analysis [90], provides another avenue for integrating caregiver perspectives and societal preferences into economic models. These approaches align with evolving HTA standards [91], supporting more equitable and evidence-based policy decisions for SMA and other rare diseases.

### Costs and resources used

Six studies [39, 41, 42, 46, 47, 53] utilised different sources to collect and quantify resource use data including RWE surveys, Hospital Episode Statistics (HES), literature reviews, NHS reference costs, expert opinions, and clinical trials data to gather information.

NICE TA588 [41] and NICE TA755 [42] used data specific to the UK healthcare system, while CADTH [46] and CADTH [47] sourced their data from studies conducted in Germany and Canada, respectively. The diversity in data sources could lead to variations in the estimation of resource use and costs, impacting the comparability of results across different studies. For instance, differences in data collection methods, such as retrospective hospital records versus prospective clinical trials, can result in inconsistent resource utilisation estimates.

Moreover, the inclusion of indirect costs, such as caregiver time or productivity losses, varies across studies, further complicating cost comparisons. This highlights the importance of ensuring that cost components and their measurements align with the specific context of the country for which the evaluation is intended to inform policy or decision-making.

Meijer et al [53] and Thokala et al. [39] adjusted costs to reflect the economic impact of SMA treatment on different age groups. The consideration of age-adjusted costs allows for a more nuanced understanding of the financial burden associated with treating SMA patients across various stages of life. Assumptions regarding costs varied across studies, influencing the projection of financial implications related to SMA treatment. For instance, some studies assumed lifelong treatment efficacy, predefined discontinuation rates, and assumptions on disease progression, caregiver disutilities, and additional costs for SMA complications. (TA588 (2019) [41], TA755 (2021) [42], CADTH (2019) [46], Meijer et al. (2023) [53], Thokala et al. (2020) [39], and CADTH (2021) [47]. The different assumptions made in each study could contribute to discrepancies in cost estimates and affect the overall economic evaluations. NICE TA588 [41] and CADTH [47] provided distinct definitions of BSC or standard of care, highlighting the variations in approaches to determine the baseline for cost-effectiveness comparisons.

Differences in defining BSC or standard of care could impact the evaluation of the incremental benefits and costs of newer treatment options for SMA. The lack of direct comparative data between different interventions (e.g., nusinersen, risdiplam, onasemnogene abeparvovec) and BSC presents a significant challenge in conducting robust cost-effectiveness analyses. This gap primarily stems from the rarity of SMA, which limits the feasibility of conducting large-scale head-to-head clinical trials comparing these treatments, alongside ethical concerns about withholding disease-modifying therapies in control groups receiving only supportive care [36, 42] Variability in assumptions, data sources, and methodological approaches among studies further complicate the comparison of resource use data and cost estimates. A key consideration regarding the costs of BSC is the source of these costs, which can vary across countries. In some settings, the majority of BSC costs are covered by the government, whereas in others, they may be borne by individuals or insurance companies. Recognising this distinction is crucial, particularly when results are presented from the perspective of governmental or public healthcare systems, such as the NHS.

The inconsistent inclusion of indirect costs in SMA economic evaluations mirrors findings from other disease areas such as multiple sclerosis [92], where societal perspectives (including productivity losses and informal care) have variable impacts on cost-effectiveness. In rare diseases, a review of 14 evaluations found that incorporating societal costs rarely changed conclusions, with only one study affected due to high treatment costs and modest QALY gains [93] Similarly, long-term indirect costs in SMA, such as parental productivity losses, may be underestimated. In contrast, in chronic adult-onset conditions like multiple sclerosis and depression, societal costs influenced results in 15 and 9% of cases, respectively, often making interventions appear more cost-effective by capturing informal care and work-related burdens [92, 94] These findings underscore the value of applying a societal perspective in SMA cost-effectiveness analyses. Although this perspective is not formally adopted in some settings (e.g., NHS and Personal Social Services (PSS) perspectives in the UK), presenting results from a societal viewpoint as a scenario analysis may be useful for illustrating its impact on ICERs.

In summary, the collection and quantification of resource use data in studies related to SMA treatment involve a variety of sources, age-adjusted costs, assumptions about costs, definitions or components of BSC, and challenging comparisons due to the complexity and heterogeneity of the disease and treatments. Interpretation of the results from these studies should consider the nuances in data sources, methodologies, and assumptions used in conducting economic evaluations for SMA management.

### Key modelling assumptions and policy implications

Economic models are inherently shaped by underlying assumptions, which may potentially influence cost-effectiveness outcomes and subsequent policy decisions [95, 96] Throughout the studies reviewed, assumptions varied considerably, particularly regarding the durability of treatment benefits. For instance, NICE TA588 (2019) [41], SMC (2018) [49], and CADTH [46–48] assumed sustained motor milestone gains, resulting in favourable QALY estimates for DMTs. In contrast, scenarios incorporating relapse showed ICERs increasing by 1.5–6-fold from base cases [34, 35] These variations underscore challenges noted in rare disease HTA, where uncertainty in long-term outcomes can lead to divergent cost-effectiveness estimates.[95, 97–99]

Utility values, especially those including caregiver disutilities, further influenced ICERs. Studies incorporating caregiver effects (e.g., NICE TA755, 2021 [42]; CADTH, 2021 [47]) reported lower ICERs than those excluding them (e.g., Malone et al., 2019 [36]). Sensitivity analyses consistently identified utility values as key ICER drivers (NICE HST24, 2023 [44]; Dean et al., 2021 [35]). These findings echo broader critiques of economic modelling in rare diseases, where heterogeneous assumptions complicate cross-study comparisons.[100, 101]

These variations carry significant policy implications. Flexible frameworks, such as NICE, enabled access to high-cost therapies like nusinersen and onasemnogene abeparvovec under managed access agreements, despite ICERs exceeding £100,000/QALY [41, 43] Conversely, more conservative approaches, as seen in CADTH evaluations (ICERs >$665,570), led to non-cost-effective conclusions and restricted access for patients [46] This divergence underscores the need for standardised modelling frameworks to enhance consistency, transparency, and equity in reimbursement decisions. Adopting harmonised approaches, such as those proposed by The Professional Society for Health Economics and Outcomes Research (ISPOR), could reduce variability and enhance decision-making reliability.[102]

### Comparison to previous related SLR

Our SLR addresses key methodological limitations identified in previous reviews on economic evaluations in SMA [10, 25–27], as assessed using the AMSTAR 2 checklist [28] These earlier reviews often lacked predefined protocols, comprehensive inclusion/exclusion criteria, risk of bias assessments, and duplicate review processes. Some were not fully systematic in scope or limited to selected data sources such as NICE reports. In contrast, our review adheres to rigorous methodological standards. We employed a transparent protocol, conducted duplicate screening and data extraction, and performed structured methodological quality assessments. Our search strategy was comprehensive, covering multiple databases and grey literature to capture all relevant studies, including those published after August 2019. We also ensured independence and transparency throughout the screening and selection process to minimise conflicts of interest.

Additionally, our review provides detailed reporting on the economic models, assumptions, outcomes, and funding sources of included studies—addressing the reporting gaps noted in prior reviews. By resolving these limitations, our SLR delivers a more robust, transparent, and up-to-date synthesis, offering a stronger foundation for health economic analysis and policy decision-making in SMA.

### Strengths and limitations

This systematic review provides important insights into the economic evaluations of DMTs for treating SMA. It provides a highly comprehensive synthesis of economic evaluations for SMA treatments, employing a rigorous search strategy across published and grey literature, following Cochrane and PRISMA guidelines [30] and employing established quality appraisal tools, including the CHEERS and Philips checklists [32, 33] The dual independent screening, data extraction, and quality assessment processes enhanced the review’s reliability and minimised bias. Additionally, the review critically appraised both published and grey literature, ensuring a broad and inclusive evidence base.

Limitations of the review include only English-language studies were included. This decision may have led to the exclusion of relevant non-English literature. We focused solely on full economic evaluations, excluding studies that reported only costs or outcomes, which could still offer useful input for economic modelling. Finally, studies evaluating the cost-effectiveness of SMA screening were excluded, despite their potential to inform aspects of economic evaluation, particularly for presymptomatic populations. These exclusions were mainly due to time and resource constraints. Overall, synthesis of the included studies was challenged by substantial heterogeneity in model structures, assumptions, and cost-effectiveness outcomes, limiting comparability. The reliance on short-term trial data to estimate long-term outcomes introduced further uncertainty, as did the frequent use of expert opinion or assumptions for key model inputs such as health utilities and treatment sustainability.

### Implications for research

Our findings demonstrate the systematic gaps in long-term data, utility measurement challenges, and modelling assumptions. Addressing these gaps requires coordinated efforts across research, clinical practice, and policy. Future studies should prioritise the collection of long-term RWE through patient registries and post-marketing surveillance to validate assumptions on treatment durability and survival. There is also a need to develop robust, patient (and caregiver) derived utility values (particularly for paediatric SMA populations) to reduce reliance on expert opinion which may, or may not be independent. Greater methodological transparency, harmonised modelling frameworks, and consistent application of sensitivity analyses would improve comparability across studies.

Collaborative efforts through international data-sharing initiatives could help overcome the inherent limitations of small SMA patient populations, ensuring that future economic models are more robust, generalisable, and aligned with the lived experiences of patients and families.

## Conclusion

This systematic literature review identified 21 studies of economic evaluations for SMA undertaken in six countries. The findings highlight the complexity and heterogeneity of economic evaluations for SMA treatments, particularly in resource use, utility estimation, and model assumptions. While tailored survival modelling strategies adapted to SMA types were evident, the robustness of evaluations was undermined by uncertainties in clinical data, heterogeneity in assumptions, and limited long-term evidence.

The assessment of resource use and cost methodologies revealed a wide range of approaches, reflecting the challenges in capturing the full spectrum of costs and health-state-related expenses in SMA treatments. Tailored survival modelling strategies, adapted to the specific SMA types, were emphasised across studies, underlining the need for personalised modelling to accurately reflect disease progression and treatment effects. However, common limitations, such as uncertainties in clinical data, data availability, and assumptions, impacted the robustness of the economic evaluations.

Future research should move beyond describing these limitations and actively address them to enhance rigour of economic evaluations. Studies should incorporate extensive sensitivity and scenario analyses, integrate real-world longitudinal data, where possible, to strengthen long-term projections, and apply transparent and standardised modelling practices. The development of internationally accepted approaches to utility measurement and resource use and cost components will also be critical to improving economic evaluations improving patient outcomes in rare diseases.

## Electronic supplementary material

Below is the link to the electronic supplementary material.

Supplementary Material 1

## Data Availability

Not applicable.

## Abbreviations

AMSTAR: A measurement tool to assess systematic reviews
BSC: Best supportive care
CADTH: Canadian Agency for Drugs and Technologies in Health
CHEERS: Consolidated Health Economic Evaluation Reporting Standards
DMTs: Disease modifying therapies
HEAP: Health economics analysis plan
HRQoL: Health-related quality of life
ICER: Incremental cost-effectiveness ratio
ISPOR: The Professional Society for Health Economics Outcomes Research
LYG: Life-years gained
NHS: National Health Service
NICE: National Institute for Health Care Excellence
PRISMA: Preferred Reporting in Systematic Reviews and Meta-analysis
PSS: Personal Social Services
QALY: Quality-adjusted life-years
RoB: Risk of Bias
RWD: Real world data
RWE: Real world evidence
SLR: Systematic literature review
SMA: Spinal muscular atrophy
SMC: Scottish Medicines Consortium
