# Supplementary material for: Economic evaluations of disease-modifying therapies for spinal muscular atrophy: a systematic literature review
Source: Orphanet J Rare Dis. 2025 Dec 5;21:10. doi: 10.1186/s13023-025-04150-z (PMC12797846; doi:10.1186/s13023-025-04150-z)
Supplement: Supplementary file 1 — Supplementary Material 1 [file 13023_2025_4150_MOESM1_ESM.docx]

**Economic evaluations of disease-modifying therapies for spinal muscular atrophy: a systematic literature review Supplementary Material**

Mehdi Yousefi,^1^ Amin Mehrabian,^1^ Anna Brown,^1^ Furqan Butt,^1^ Jeremiah Donoghue,^1^ Janette Parr,^1^ Mubarak Patel,^1^ Amy Grove,^1^ Jo Parsons,^1^ Peter Auguste^1^

^1^Centre for Evidence and Implementation Science, Health Services Management Centre, University of Birmingham, Birmingham, B15 2RT

Correspondence to: Dr Peter Auguste

Centre for Evidence and Implementation Science

Health Services Management Centre

School of Social Policy and Society

College of Social Sciences

University of Birmingham

Birmingham, B15 2RT

Email: p.e.auguste.1@bham.ac.uk

Declaration of funding

This study was funded by the NIHR Evidence Synthesis Programme as project number NIHR136129.

**Appendix 1: Critical appraisal of previous systematic reviews**

**Table 1: AMSTAR 2; a critical appraisal tool for systematic reviews that include randomised or nonrandomised studies of healthcare interventions, or both**

| First author | **Paracha N (2022) [1]** | **Wiedmann L (2023) [2]** | **Yang M (2022) [3]** | **Dangouloff T (2021) [4]** |
| --- | --- | --- | --- | --- |
| Journal | Pharmacoeconomics | Expert Review of Pharmacoeconomics & Outcomes Research | Advances in Therapy | Orphanet Journal of Rare Diseases |
| Study title | Systematic Literature Review to Assess Economic Evaluations in Spinal Muscular Atrophy (SMA). | Review of economic modeling evidence from NICE appraisals of rare disease treatments for spinal muscular atrophy | Systematic Literature Review of Clinical and Economic Evidence for Spinal Muscular Atrophy | Systematic literature review of the economic burden of spinal muscular atrophy and economic evaluations of treatments |
| Co-authors | Hudson P, Mitchell S, Sutherland CS | Cairns J | Awano H, Tanaka S, Toro W, Zhang S, Dabbous O, Igarashi A. | Botty C, Beaudart C, Servais L, Hiligsmann M |
| Source of publication  Journal yy;vol(issue):pp | Apr;40(Suppl 1):69-89 | Jun;23(5):469-482 | May;39(5):1915-1958 | Jan 23;16(1):47 |
| Language | English | English | English | English |
| Publication type | Systematic Literature Review | in-depth review (does NOT contain more detail about study type) | Systematic Literature Review | Systematic Literature Review |
| 1. Did the research questions and inclusion criteria for the review include the components of PICO? | Yes  The authors stated that there were two main objectives. First, to identify the modelling approaches used to assess current approved treatments relevant to SMA. Second, to identify economic evaluations that assessed other neuromuscular disorders | Yes  To examine the different modelling approaches for SMA by comparing and discussing issues across economic evaluations of three appraisals submitted to NICE. | Yes  To summarise the literature on the natural history of SMA, and the impact of disease modifying therapies on SMA, HRQoL, clinical efficacy and safety and economic impact | No  “In this study, we systematically review the economic burden of SMA (in terms of costs) and provide an overview and critical appraisal of economic evaluations in SMA.” |
| 2. Did the report of the review contain an explicit statement that the review methods were established prior to the conduct of the review and did the report justify any significant deviations from the protocol? | No  It does Not contain any statement for review question(s)/ a search strategy/ inclusion/exclusion criteria or a risk of bias assessment | No | No  It does Not contain any statement for review question(s)/ a search strategy/ inclusion/exclusion criteria or a risk of bias assessment | No  It does Not contain any statement for review question(s)/ a search strategy/ inclusion/exclusion criteria or a risk of bias assessment |
| 3. Did the review authors explain their selection of the study designs for inclusion in the review? | Yes  Without any limitation for including both RCTs and NRSI | Yes  The documents available for the NICE appraisals of the three rare disease treatments (RDTs) for SMA were reviewed. | Yes  Without any limitation for including both RCTs and NRSI | Yes  It seems there were NOT limitations for including both RCTs and NRSI |
| 4. Did the review authors use a comprehensive literature search strategy? | Partial Yes  searched at least 2 databases AND provided key word and/or search strategy AND justified publication restrictions (e.g. language) | No  Relevant documents for each appraisal include the final scope, the company evidence submission, the report by the External Assessment Group (EAG), the final appraisal or evaluation document, and documents relating to respective managed access agreements (MAAs), are all publicly available and were retrieved from the NICE website. | Yes   - searched at least 2 databases AND provided key word and/or search strategy AND justified publication restrictions (e.g. language) - searched the reference lists / bibliographies of included studies - searched trial/study registries | Partial Yes  searched at least 2 databases AND provided key word and/or search strategy AND justified publication restrictions (e.g. language) |
| 5. Did the review authors perform study selection in duplicate? | No  Screening of titles/abstracts appeared to be undertaken by one reviewer. | No | Yes  two reviewers independently agreed on selection of eligible studies | Yes  Two researchers first screened titles and abstracts independently for eligibility and then evaluated the full text. |
| 6. Did the review authors perform data extraction in duplicate? | Yes  at least two reviewers achieved consensus on which data to extract from included studies | No  Data was extracted by a single person (LW) which may be a potential limitation. | No | No |
| 7. Did the review authors provide a list of excluded studies and justify the exclusions? | No | No | No | No |
| 8. Did the review authors describe the included studies in adequate detail? | Partial Yes,  Authors have provided details about some of the characteristics of the economic analyses (e.g., intervention and comparators, model types, model outcomes, perspectives, discounting, and time horizon)  Reported that studies undertook analyses to address uncertainty but have not provided these results. | Partial Yes,  details were extracted for five categories: 1) the economic model, 2)survival modelling, 3) cost and healthcare resource use, 4) measurement and valuation of health effects, and 5) the committee recommendation. | Partial Yes,  the study described: populations, interventions, comparators, outcomes  Presented information based on SMA types and median age of onset from individual studies.  Provided an overview and explanation of the overall results from studies included in the systematic review.  And NOT described:  research designs, incremental cost/QALYs, ICER,  Little details are provided on the conduct of economic analyses. Not many details from individual studies about the characteristics of studies. | Partial Yes,  the study described: populations, interventions, comparators, outcomes and research designs  But Not sufficient details about health states and transitions between these health states.  Lot of focus on costs and not on other aspects. What is the age of the starting population? What discount rates were used? |
| 9. Did the review authors use a satisfactory technique for assessing the risk of bias (RoB) in individual studies that were included in the review? | No  The study has NOT contained the assessment of risk of bias (RoB) for different types of studies (**RCTs or NRSI)** | No | Partial Yes  selection of the reported result from among multiple measurements or analyses of a specified outcome  Did not appear to critically appraise the studies that undertook an economic analysis | Partial Yes  from selection bias  To limit the possibility of biased results, two reviewers independently reviewed the quality appraisal of the included studies. |
| 10. Did the review authors report on the sources of funding for the studies included in the review? | No  Have NOT reported on the sources of funding for individual studies included in the review. | Yes | No | No |
| 11. If meta-analysis was performed did the review authors use appropriate methods for statistical combination of results? | No meta-analysis conducted  (NOT for **RCTs or NRSI)** | No meta-analysis conducted | No meta-analysis conducted | No meta-analysis conducted |
| 12. If meta-analysis was performed, did the review authors assess the potential impact of RoB in individual studies on the results of the meta-analysis or other evidence synthesis? | No meta-analysis conducted | No meta-analysis conducted | No meta-analysis conducted | No meta-analysis conducted |
| 13. Did the review authors account for RoB in individual studies when interpreting/ discussing the results of the review? | Yes  But the results is not reported | No | Yes | Yes  Although the number of studies is too limited to make reliable comparison between industry-sponsored and non-industry sponsored economic evaluations, and the fact that no relationship was observed in other diseases this remains a potential study publication bias as pharmaceutical companies could tend to present most favourable results. |
| 14. Did the review authors provide a satisfactory explanation for, and discussion of, any heterogeneity observed in the results of the review? | Yes,  Authors included a discussion about emerging economic evaluations in SMA, implementation of HTA body recommendations, long-term modelling implications, consistency in appropriate motor function assessment, consensus in modelling structure and SMA health state classification and measuring change that is meaningful for patients in economic evaluations, which are all important when considering the modelling methods to assess DMTs for treating SMA. | Yes  The authors concluded that there is an increased need for consistency in the economic modelling of DMTs for treatment for SMA. Additionally, further analyses (clinical and economic) could focus on how new evidence collected from the MAA reduce uncertainty in the economic modelling | Yes  Authors found that there was methodological heterogeneity between studies included in the systematic review.  They concluded that there is a clear need for up-to-date and methodologically rigorous and clinical, HRQoL, and economic data to support unbiased assessments of the cost-effectiveness of future SMA treatments. | No  Didn’t Discuss the variations in the range of results reported. |
| 15- If they performed quantitative synthesis did the review authors carry out an adequate investigation of publication bias (small study bias) and discuss its likely impact on the results of the review? | No meta-analysis conducted | No meta-analysis conducted | No meta-analysis conducted | No meta-analysis conducted |
| 16. Did the review authors report any potential sources of conflict of interest, including any funding they received for conducting the review? | Yes  In the 'Conflict of Interest' section, the study merely mentioned the author's relationship with Roche. However, the authors did not clearly report any competing interests. Consequently, the possibility of a conflict of interest exists. | Yes  The authors have no other relevant affiliations or financial involvement with any organization or entity with a financial interest in or financial conflict with the subject matter or materials discussed in the manuscript apart from those disclosed. | Yes  In the Disclosures' section, the study merely mentioned the author's relationship with NOVARTIS PHARMA K.K. However, the authors did not clearly report any competing interests. Consequently, the possibility of a conflict of interest exists. | Yes  Some of authors had have some lectures that sponsored by Biogen and Roche or they give lectures and has served as a consultant for Roche, Biogen, Avexis, and Cytokinetics. |
| General comments | 1. Study Published online: 18 October 2021 2. comprehensive search was performed on 29 August 2019 with a different start date (for example: Embase from 1974, MEDLINE from 1946). 3. Additional searches (hand searching) of congress proceedings from the past 3 years (2017–2019) were conducted. | 1. The manuscript was received by the journal on October 3, 2022. 2. This review was NOT categorized as a systematic review (SR). 3. This review is a detailed analysis of NICE appraisals for SMA and aims to compare the economic modeling evidence from the three RDTs. | 1. The manuscript was received by the journal on December 9, 2021. 2. Literature reviews were performed on November 12–13, 2020 | 1. A systematic review of the literature in PubMed and Scopus up to 15 September 2020 was conducted according to PRISMA guidelines. 2. The study searched for original, full-text articles reporting costs or economic evaluations of SMA published after January 1, 1998. 3. Costs and ICERs were converted to 2020 US dollars to facilitate comparison 4. The initial searches (conducted in December 2019) |
| Strengths | 1. The primary objective of this SLR was to identify the modelling approaches in economic evaluations 2. Some sections of individual studies, such as the model structure and results, are reported completely. 3. The study has conducted a comprehensive search strategy | 1. This study was conducted as an in-depth review of three main treatments for SMA 2. It offers a comprehensive comparison of the economic modeling data presented in the three RDTs. 3. The study collected and analyzed relevant information on the three RDTs from various reports within the NICE organization. These reports include the final scope, the company's evidence submission, the report by the External Assessment Group (EAG), the final appraisal or evaluation document, and documents related to managed access agreements (MAAs). | 1. The research explored various facets of MSA. 2. It utilized a strong methodology. 3. The study employed a comprehensive search strategy. | 1. Methodological quality of economic analysis appraised against the CHEC-extended tool. 2. the review authors describe the included studies in adequate detail. It States sufficient information on the conduct of the study, but other information could have been reported. 3. the searches performed in two time (December 2019 and September 2020) |
| limitations/concerns | 1. The method of the study has some weaknesses. 2. The inclusion and exclusion criteria were not clearly reported. For example, it is not clear which items were essential for inclusion, and some filters for excluding certain types of studies are not clearly mentioned. 3. The quality assessment of included economic evaluations was conducted for five studies (table 6), but according to figure 1 (PRISMA flow diagram), the total number of studies included in the systematic literature review is nine. | 1. This study was just about related reports in NICE and was NOT conducted as a systematic review (SR). 2. This study did NOT report the incremental costs/QALYs and ICER. 3. This study did NOT report the assumptions of each study. 4. It did NOT provide any quality assessment of the included studies. 5. The method section of the study is very abstract and did NOT provide details about the study process. | 1. The study attempted to cover a broad range of information regarding various aspects of SMA, but this resulted in a superficial review. 2. The economic modeling of the included studies was not addressed in the study. 3. We do not have access to the search strategy algorithm or the completed checklist for quality assessment. | 1. The search strategy isn’t strong. 2. The study didn’t cover some databases and websites regarding HTA reports. 3. The original data about costs and ICER were converted to $US and we can’t access that data. 4. Would have liked to see a comparison with the key modelling assumptions made, especially with regards to the long-term effectiveness of treatment. Did any model include waning of the treatment effect? |
| Summary of the reviewers(two reviewers) | Systematic Literature Review is a Critically Low quality review | As a review only focusing on some reports in the NICE, it may be considered adequate. However, when evaluated against the criteria of a systematic literature review, it falls into the category of low quality. | Systematic Literature Review is a Critically Low quality review | Systematic Literature Review is a Critically Low quality review |
| Do we need new SLR? | Yes, for some reasons:   1. New related studies published after August 2019. 2. New SLR must be conducted clearly with no conflict of interest. 3. We need an SLR with a robust methodology, particularly focusing on RoB (Risk of Bias), inclusion and exclusion criteria, and study protocol. | Yes, for some reasons:  We're in need of a systematic literature review (SLR), and it's crucial to mention that this specific study falls short of meeting the necessary criteria for an SLR. For instance, it lacks elements like a thorough search strategy, quality assessment and inclusion and exclusion criteria. | Yes, for some reasons:  We require a systematic literature review (SLR) that presents solid evidence regarding the model structure and results of each individual study. | Yes, for some reasons:  We need a comprehensive SLR.  New related studies published after August 2020. |

**Appendix 2:** **Literature search strategies**

Table 2: Overview of literature searches undertaken

| *Cost effectiveness review: bibliographic databases* | | |
| --- | --- | --- |
| **Database** | **Date searched** | **Number of records** |
| Embase (Ovid) | 30/01/24 | 514 |
| MEDLINE All (Ovid) | 30/01/24 | 116 |
| Science Citation Index and Conference Proceedings (Web of Science) | 31/01/24 | 195 |
| International HTA database (INAHTA) | 30/01/24 | 33 |
| CEA Registry (Tufts Medical Center) | 30/01/24 | 13 |
| EconPapers (RePec) | 30/01/24 | 9 |
| **Total number of records retrieved: 880**  **Duplicates removed (EndNote): 220**  **Final number for screening: 660** | | |
| *Cost effectiveness review: other sources* | | |

| Internet (Google) | 31/01/24 | 9 |
| --- | --- | --- |
| Websites (NICE, SMC, CADTH, ICER, FDA, MHRA, EMA) | 25/01/24 | 31 documents + 1 web page (ongoing project) from 21 projects/reviews |
| **Total number sought for retrieval: 41**  **Reports not retrieved/available: 0**  **Duplicates removed: 7**  **Final number for screening: 34** | | |

| *Update search: bibliographic databases* | | |
| --- | --- | --- |
| **Database** | **Date searched** | **Number of records** |
| Embase (Ovid) | 10/02/25 | 613 |
| MEDLINE All (Ovid) | 10/02/25 | 141 |
| Science Citation Index and Conference Proceedings (Web of Science) | 10/02/25 | 238 |
| International HTA database (INAHTA) | 10/02/25 | 41 |
| CEA Registry (Tufts Medical Center) | 10/02/25 | 14 |
| EconPapers (RePec) | 10/02/25 | 15 |
| **Total number of records retrieved: 1,062**  **Duplicates removed (both within this set and against original search results) (EndNote): 924**  **Final number for screening: 138** | | |

| *Update search: Other sources* | | |
| --- | --- | --- |
| Internet (Google) | 11/02/25 | 1 |
| Websites (NICE, SMC, CADTH, ICER, FDA, MHRA, EMA) | 11/02/25 | 0 |
| **Total number sought for retrieval: 1**  **Reports not retrieved/available: 0**  **Final number for screening: 1** | | |

**Search strategies: cost-effectiveness review**

Search strategies: original search

**Embase (Ovid)**

Date searched: 30/01/24

Embase <1974 to 2024 January 29>

1 exp hereditary spinal muscular atrophy/ or spinal muscular atrophy/ 14240

2 (spinal muscul* atroph* or SMA).kf,tw. 50753

3 (Werdnig adj Hoffman*).kf,tw. 423

4 (Kugelberg adj Welander*).kf,tw. 216

5 1 or 2 or 3 or 4 [disease terms] 55889

6 nusinersen/ 1915

7 (nusinersen or spinraza* or isis smn* or isis 396443).kf,tn,tw. 1701

8 risdiplam/ 567

9 (risdiplam or evrysdi* or RG 7916 or RG7916).kf,tn,tw. 485

10 onasemnogene abeparvovec/ 928

11 (onasemnogene or zolgensma* or AVXS-101).kf,tn,tw. 850

12 6 or 7 or 8 or 9 or 10 or 11 2860

13 Economics/ 245379

14 Cost/ 63913

15 exp Health Economics/ 1055902

16 Budget/ 34254

17 budget*.ti,ab,kw. 48761

18 (economic* or cost or costs or costly or costing or price or prices or pricing or pharmacoeconomic* or pharmaco-economic* or expenditure or expenditures or expense or expenses or financial or finance or finances or financed).ti,kw. 322778

19 (economic* or cost or costs or costly or costing or price or prices or pricing or pharmacoeconomic* or pharmaco-economic* or expenditure or expenditures or expense or expenses or financial or finance or finances or financed).ab. /freq=2 553765

20 (cost* adj2 (effective* or utilit* or benefit* or minimi* or analy* or outcome or outcomes)).ab,kw. 295295

21 (value adj2 (money or monetary)).ti,ab,kw. 4226

22 Statistical Model/ 176325

23 economic model*.ab,kw. 6445

24 Probability/ 151208

25 markov.ti,ab,kw. 37781

26 monte carlo method/ 52474

27 monte carlo.ti,ab,kw. 63410

28 Decision Theory/ 1861

29 Decision Tree/ 23267

30 (decision* adj2 (tree* or analy* or model*)).ti,ab,kw. 54152

31 or/13-30 [Economic Evaluations & Models - Embase. In: CADTH Search Filters Database. Ottawa: CADTH; 2023: https://searchfilters.cadth.ca/link/15] 2053618

32 socioeconomics/ 164905

33 exp Quality of Life/ 676846

34 quality of life.ti,kw. 179076

35 ((instrument or instruments) adj3 quality of life).ab. 5526

36 Quality-Adjusted Life Year/ 36559

37 quality adjusted life.ti,ab,kw. 27304

38 (qaly* or qald* or qale* or qtime* or life year or life years).ti,ab,kw. 46217

39 disability adjusted life.ti,ab,kw. 6856

40 daly*.ti,ab,kw. 6736

41 (sf36 or sf 36 or short form 36 or shortform 36 or short form36 or shortform36 or sf thirtysix or sfthirtysix or sfthirty six or sf thirty six or shortform thirtysix or shortform thirty six or short form thirtysix or short form thirty six).ti,ab,kw. 51145

42 (sf6 or sf 6 or short form 6 or shortform 6 or sf six or sfsix or shortform six or short form six or shortform6 or short form6).ti,ab,kw. 3036

43 (sf8 or sf 8 or sf eight or sfeight or shortform 8 or shortform 8 or shortform8 or short form8 or shortform eight or short form eight).ti,ab,kw. 1047

44 (sf12 or sf 12 or short form 12 or shortform 12 or short form12 or shortform12 or sf twelve or sftwelve or shortform twelve or short form twelve).ti,ab,kw. 12605

45 (sf16 or sf 16 or short form 16 or shortform 16 or short form16 or shortform16 or sf sixteen or sfsixteen or shortform sixteen or short form sixteen).ti,ab,kw. 71

46 (sf20 or sf 20 or short form 20 or shortform 20 or short form20 or shortform20 or sf twenty or sftwenty or shortform twenty or short form twenty).ti,ab,kw. 537

47 (hql or hqol or h qol or hrqol or hr qol).ti,ab,kw. 40632

48 (hye or hyes).ti,ab,kw. 189

49 (health* adj2 year* adj2 equivalent*).ti,ab,kw. 53

50 (pqol or qls).ti,ab,kw. 764

51 (quality of wellbeing or quality of well being or index of wellbeing or index of well being or qwb).ti,ab,kw. 620

52 nottingham health profile*.ti,ab,kw. 1688

53 nottingham health profile/ 670

54 sickness impact profile.ti,ab,kw. 1299

55 sickness impact profile/ 2405

56 health status indicator/ 3549

57 (health adj3 (utilit* or status)).ti,ab,kw. 122035

58 (utilit* adj3 (valu* or measur* or health or life or estimat* or elicit* or disease or score* or weight)).ti,ab,kw. 26395

59 (preference* adj3 (valu* or measur* or health or life or estimat* or elicit* or disease or score* or instrument or instruments)).ti,ab,kw. 19792

60 disutilit*.ti,ab,kw. 1323

61 rosser.ti,ab,kw. 141

62 willingness to pay.ti,ab,kw. 13671

63 standard gamble*.ti,ab,kw. 1226

64 (time trade off or time tradeoff).ti,ab,kw. 2452

65 tto.ti,ab,kw. 2300

66 (hui or hui1 or hui2 or hui3).ti,ab,kw. 3241

67 (eq or euroqol or euro qol or eq5d or eq 5d or euroqual or euro qual).ti,ab,kw. 39842

68 duke health profile.ti,ab,kw. 121

69 functional status questionnaire.ti,ab,kw. 180

70 dartmouth coop functional health assessment*.ti,ab,kw. 14

71 or/32-70 [ Economic - Health Utilities / Quality of Life - Standard - Embase. In: CADTH Search Filters Database. Ottawa: CADTH; 2022: https://searchfilters.cadth.ca/link/18.] 1025897

72 5 and 12 and 31 390

73 5 and 12 and 71 235

74 72 or 73 514

The Embase search strategy was peer reviewed by Naila Dracup, Information Specialist, Warwick Medical School.

Lines 13-31 and 32-72 are search filters developed by CADTH:

Economic Evaluations & Models - Embase. In: CADTH Search Filters Database. Ottawa: CADTH; 2024: <https://searchfilters.cadth.ca/link/15> . Accessed 2024-01-30, and

Economic - Health Utilities / Quality of Life - Standard - Embase. In: CADTH Search Filters Database. Ottawa: CADTH; 2024: [https://searchfilters.cadth.ca/link/18 . Accessed 2024-01-30](https://searchfilters.cadth.ca/link/18%20.%20Accessed%202024-01-30).

**MEDLINE (Ovid)**

Date searched: 30/01/24

Ovid MEDLINE(R) ALL <1946 to January 29, 2024>

1 exp Muscular Atrophy, Spinal/ 6595

2 (spinal muscul* atroph* or SMA).mp. 33145

3 (Werdnig adj Hoffman*).mp. 399

4 (Kugelberg adj Welander*).mp. 210

5 1 or 2 or 3 or 4 [disease terms] 34989

6 (nusinersen or spinraza* or isis smn* or isis 396443).mp. 773

7 (risdiplam or evrysdi* or RG 7916 or RG7916).mp. 164

8 (onasemnogene or zolgensma* or AVXS-101).mp. 269

9 6 or 7 or 8 [intervention or comparator terms] 961

10 Economics/ 27523

11 exp "Costs and Cost Analysis"/ 268504

12 Economics, Nursing/ 4013

13 Economics, Medical/ 9269

14 Economics, Pharmaceutical/ 3126

15 exp Economics, Hospital/ 25795

16 Economics, Dental/ 1921

17 exp "Fees and Charges"/ 31454

18 exp Budgets/ 14187

19 budget*.ti,ab,kf. 37307

20 (economic* or cost or costs or costly or costing or price or prices or pricing or pharmacoeconomic* or pharmaco-economic* or expenditure or expenditures or expense or expenses or financial or finance or finances or financed).ti,kf. 290781

21 (economic* or cost or costs or costly or costing or price or prices or pricing or pharmacoeconomic* or pharmaco-economic* or expenditure or expenditures or expense or expenses or financial or finance or finances or financed).ab. /freq=2 397747

22 (cost* adj2 (effective* or utilit* or benefit* or minimi* or analy* or outcome or outcomes)).ab,kf. 219322

23 (value adj2 (money or monetary)).ti,ab,kf. 3152

24 exp models, economic/ 16261

25 economic model*.ab,kf. 4374

26 markov chains/ 16079

27 markov.ti,ab,kf. 30218

28 monte carlo method/ 32637

29 monte carlo.ti,ab,kf. 62092

30 exp Decision Theory/ 13553

31 (decision* adj2 (tree* or analy* or model*)).ti,ab,kf. 40912

32 or/10-31 [Economic Evaluations & Models - MEDLINE. In: CADTH Search Filters Database. Ottawa: CADTH; 2024: https://searchfilters.cadth.ca/link/16] 929288

33 "Value of Life"/ 5821

34 Quality of Life/ 281310

35 quality of life.ti,kf. 121344

36 ((instrument or instruments) adj3 quality of life).ab. 3996

37 Quality-Adjusted Life Years/ 16108

38 quality adjusted life.ti,ab,kf. 18209

39 (qaly* or qald* or qale* or qtime* or life year or life years).ti,ab,kf. 29608

40 Disability-Adjusted Life Years/ 227

41 disability adjusted life.ti,ab,kf. 5837

42 Healthy Life Expectancy/ 71

43 (daly* or disability free life expectanc* or haly* or health* life expectanc*).ti,ab,kf. 6974

44 (sf36 or sf 36 or short form 36 or shortform 36 or short form36 or shortform36 or sf thirtysix or sfthirtysix or sfthirty six or sf thirty six or shortform thirtysix or shortform thirty six or short form thirtysix or short form thirty six).ti,ab,kf. 31483

45 (sf6 or sf 6 or short form 6 or shortform 6 or sf six or sfsix or shortform six or short form six or shortform6 or short form6).ti,ab,kf. 2723

46 (sf8 or sf 8 or sf eight or sfeight or shortform 8 or shortform 8 or shortform8 or short form8 or shortform eight or short form eight).ti,ab,kf. 640

47 (sf12 or sf 12 or short form 12 or shortform 12 or short form12 or shortform12 or sf twelve or sftwelve or shortform twelve or short form twelve).ti,ab,kf. 7960

48 (sf16 or sf 16 or short form 16 or shortform 16 or short form16 or shortform16 or sf sixteen or sfsixteen or shortform sixteen or short form sixteen).ti,ab,kf. 41

49 (sf20 or sf 20 or short form 20 or shortform 20 or short form20 or shortform20 or sf twenty or sftwenty or shortform twenty or short form twenty).ti,ab,kf. 467

50 (hql or hqol or h qol or hrqol or hr qol).ti,ab,kf. 25339

51 (hye or hyes).ti,ab,kf. 77

52 (health* adj2 year* adj2 equivalent*).ti,ab,kf. 48

53 (pqol or qls).ti,ab,kf. 476

54 (quality of wellbeing or quality of well being or index of wellbeing or index of well being or qwb).ti,ab,kf. 509

55 nottingham health profile*.ti,ab,kf. 1249

56 sickness impact profile.ti,ab,kf. 1100

57 exp health status indicators/ 344631

58 (health adj3 (utilit* or status)).ti,ab,kf. 95721

59 (utilit* adj3 (valu* or measur* or health or life or estimat* or elicit* or disease or score* or weight)).ti,ab,kf. 16607

60 (preference* adj3 (valu* or measur* or health or life or estimat* or elicit* or disease or score* or instrument or instruments)).ti,ab,kf. 15044

61 disutilit*.ti,ab,kf. 658

62 rosser.ti,ab,kf. 109

63 willingness to pay.ti,ab,kf. 9157

64 standard gamble*.ti,ab,kf. 918

65 (time trade off or time tradeoff).ti,ab,kf. 1695

66 tto.ti,ab,kf. 1458

67 (hui or hui1 or hui2 or hui3).ti,ab,kf. 2049

68 (eq or euroqol or euro qol or eq5d or eq 5d or euroqual or euro qual).ti,ab,kf. 23932

69 duke health profile.ti,ab,kf. 94

70 functional status questionnaire.ti,ab,kf. 133

71 dartmouth coop functional health assessment*.ti,ab,kf. 14

72 or/33-71 [Economic - Health Utilities / Quality of Life - MEDLINE. In: CADTH Search Filters Database. Ottawa: CADTH; 2024: https://searchfilters.cadth.ca/link/19. Accessed 2024-01-26.] 775631

73 5 and 9 and 32 70

74 5 and 9 and 72 69

75 73 or 74 116

**Science Citation Index and Conference Proceedings (Web of Science)**

Date searched: 31/01/24

Database: Web of Science Core Collection

Editions searched/Entitlements: WOS.SCI: 1970 to 2024, WOS.ISTP: 1990 to 2024

| # | Search Query | Results |
| --- | --- | --- |
| 1 | TS=(nusinersen OR spinraza* OR "isis smn*" OR "isis 396443") | 1232 |
| 2 | TS=(risdiplam OR evrysdi* OR "RG 7916" OR RG7916) | 257 |
| 3 | TS=(onasemnogene or zolgensma* or "AVXS-101") | 435 |
| 4 | #1 OR #2 OR #3 | 1653 |
| 5 | TS=(("spinal muscul*" NEAR/0 atroph*) OR SMA) | 49264 |
| 6 | TS=(Werdnig NEAR/0 Hoffman*) | 413 |
| 7 | TS=(Kugelberg NEAR/0 Welander*) | 113 |
| 8 | #5 OR #6 OR #7 | 49435 |
| 9 | TS=(cost* or economic* or pharmacoeconomic* or pharmaco-economic* or price or prices or pricing or expenditure* or expense* or financial or finance or finances or financed or budget* or (value NEAR/1 (money OR monetary)) or (economic NEAR/1 model*) or markov or monte carlo or (decision NEAR/1 (tree* or analy* or model*))) | 3676686 |
| 10 | TS=("value of life" or "quality of life" or qol or hrql or hrqol or (("quality adjusted life" OR "disability adjusted life") NEAR/0 year*) or qaly* or qald* or qale* or qtime* or "life year" or "life years" or daly* or ("disability free life" NEAR/0 expectanc*) or haly* or (health* NEAR/0 ("life expectance" or "life expectancy") or icer or "euro-qol" or euroqual or "euro qual" or utilit* or disutilit* or (net NEAR/0 benefit*) or (contingent NEAR/0 valuation*) or (preference* NEAR/2 (valu* or measur* or health or life or estimat* or elicit* or disease or score* or instrument or instruments)) or euroqol or "euro qol" or eq5d or eq-5d or "short-form 36" or "shortform 36" or sf-36 or sf36 or sf-6d or sf6d or sf-12 or sf12 or "health utilities index" or hui or hui1 or hui2 or hui3 or (time NEAR/0 trade*) or tto or "standard gamble" or sg or markov or (decision NEAR/1 model*) or (visual NEAR/0 analog*) or "discrete choice" or ((health* NEAR/0 year*) NEAR/0 equivalen*) or (health NEAR/0 stat*) or (willing* NEAR/1 pay) or resource* or wellbeing or well-being)) | 2483907 |
| 11 | #4 AND #8 AND #9 | 107 |
| 12 | #4 AND #8 AND #10 | 132 |
| 13 | #11 OR #12 | 195 |

**International HTA database (INAHTA)** <https://database.inahta.org/>

Date searched: 30/01/24

(("Muscular Atrophy, Spinal"[mhe]) OR ("spinal muscular atrophy" OR "spinal muscular atrophies" OR SMA OR "Werdnig Hoffman" OR "Kugelberg Welander"))

33 results

**CEA Registry (Tufts Medical Center)** <https://cear.tuftsmedicalcenter.org/>

Date searched: 30/01/24

Advanced search screen, Methods:

Keyword is spinal muscular atrophy

OR Keyword is SMA 14 results (1 duplicate)

Keyword is nusinersen

OR Keyword is spinraza

OR Keyword is risdiplam

OR Keyword is evrysdi

OR Keyword is onasemnogene

OR Keyword is zolgensma 8 results (all already identified above)

Total unique results: 13

**EconPapers (RePEc)** <https://econpapers.repec.org/scripts/search.pf>

Date searched: 30/01/24

Advanced search screen; Free text search box

nusinersen OR spinraza OR risdiplam OR evrysdi OR onasemnogene OR zolgensma 9 results

**Internet (Google)** <https://www.google.co.uk/>

Date searched: 31/01/24

*Records were retrieved (added to EndNote library) only if potentially relevant to the review, and not already found via the database/other searches*

economic evaluation nusinersen OR risdiplam OR onasemnogene OR spinraza OR evrysdi OR zolgensma browsed first 50 results; 8 records retrieved.

cost effectiveness nusinersen OR risdiplam OR onasemnogene OR spinraza OR evrysdi OR zolgensma browsed first 50 results; 1 record retrieved.

health technology assessment nusinersen OR risdiplam OR onasemnogene OR spinraza OR evrysdi OR zolgensma browsed first 50 results; 0 records retrieved.

Total: 9 records retrieved.

**Websites**

Date searched: 25/01/24

**National Institute for Health and Care Excellence (NICE)** <https://www.nice.org.uk/guidance/published?sp=on>

*Search for published guidance*

*Filter: Type: Guidance*

Filter by title or keyword:

Spinal muscular atrophy 4 results

- <https://www.nice.org.uk/guidance/ta755> 6 documents downloaded
- <https://www.nice.org.uk/guidance/hst15> 1 document downloaded
- <https://www.nice.org.uk/guidance/hst24> 1 document downloaded
- <https://www.nice.org.uk/guidance/ta588> 2 documents downloaded

SMA 0 results

nusinersen 1 result (already identified above)

risdiplam 1 result (already identified above)

onasemnogene 2 results (already identified above)

Total: 4 technology assessments identified; 10 potentially relevant documents downloaded

**Scottish Medicines Consortium (SMC)** <https://www.scottishmedicines.org.uk/>

*Search box on homepage*

spinal muscular atrophy 6 results, of which 3 relevant

- <https://www.scottishmedicines.org.uk/medicines-advice/nusinersen-spinraza-fullsubmission-131818/>
- <https://www.scottishmedicines.org.uk/medicines-advice/risdiplam-evrysdi-full-smc2401/>
- <https://www.scottishmedicines.org.uk/medicines-advice/onasemnogene-abeparvovec-zolgensma-full-smc2311/>

nusinersen 2 results (already identified above)

risdiplam 2 results (already identified above)

onasemnogene 2 results (already identified above)

Total: 3 technology assessments identified; 3 potentially relevant documents downloaded

**CADTH: Canada’s Drug and Health Technology Agency** <https://www.cadth.ca/search>

*All searches limited to ‘Reports’*

Spinal muscular atrophy 23 results, of which 8 projects potentially relevant:

- Nusinersen, reimbursement review 2022 <https://www.cadth.ca/nusinersen-1> (combined clinical and pharmacoeconomic review)
- Health Technology Assessment Recommendations and Managed Entry Agreements Related to Optimizing the Treatment for Pediatric Spinal Muscular Atrophy. *In progress but effective finish date was January 2023 so keep an eye out* <https://www.cadth.ca/health-technology-assessment-recommendations-and-managed-entry-agreements-related-optimizing>
- Risdiplam, reimbursement review 2021 <https://www.cadth.ca/risdiplam> (combined clinical and pharmacoeconomic review)
- Onasemnogene abeparvovec, Reimbursement review 2021 <https://www.cadth.ca/onasemnogene-abeparvovec>
  - clinical report
  - pharmacoeconomic report
- Nusinersen for Adolescents and Adults with Spinal Muscular Atrophy: A Review of Clinical Effectiveness. Rapid review 2020. <https://cadth.ca/nusinersen-adolescents-and-adults-spinal-muscular-atrophy-review-clinical-effectiveness>
- Nusinersen, reimbursement review 2018. <https://www.cadth.ca/nusinersen>
  - clinical report
  - pharmacoeconomic report
- Nusinersen for Adults with Spinal Muscular Atrophy: Clinical Effectiveness. Rapid response report; reference list. 2020. <https://www.cadth.ca/nusinersen-adults-spinal-muscular-atrophy-clinical-effectiveness>
- Nusinersen, reimbursement review 2019. <https://www.cadth.ca/nusinersen-0>
  - clinical report
  - pharmacoeconomic report

SMA 17 results, of which 0 relevant and not already identified above

nusinersen 10 results (all already identified above)

risdiplam 3 results (already identified above)

onasemnogene 6 results (already identified above)

Total: 8 projects identified; 10 potentially relevant reports downloaded, 1 ongoing project web page bookmarked

**Institute for Clinical and Economic Review (ICER)** <https://icer.org/explore-our-research/assessments/>

*search by keyword; no filters applied*

spinal muscular atrophy 1 result

- An assessment of Onasemnogene Abeparvovec and Nusinersen for Spinal Muscular Atrophy (SMA). 2019 <https://icer.org/assessment/spinal-muscular-atrophy-2019/> (evidence report includes clinical and cost effectiveness)

SMA 10 results, of which 0 relevant and not already identified above

nusinersen 1 result (already identified above)

risdiplam 0 results

onasemnogene 1 result (already identified above)

Total: 1 technology assessment identified; 1 report downloaded

**Drugs@FDA , U.S. Food & Drug Administration** <https://www.accessdata.fda.gov/scripts/cder/daf/>

nusinersen 1 review found, approval date 2016: <https://www.accessdata.fda.gov/drugsatfda_docs/nda/2016/209531Orig1s000TOC.cfm>

(separate medical, pharmacology, statistical, etc review documents but no pharmacoeconomic / cost effectiveness data). Medical review downloaded.

risdiplam 1 review found, approval date 2020: <https://www.accessdata.fda.gov/drugsatfda_docs/nda/2020/213535Orig1s000TOC.cfm> (separate clincial, statistical, etc review documents but no pharmacoeconomic / cost effectiveness data). Clinical review downloaded.

onasemnogene 0 results

zolgensma 0 results

Total: 2 drug reviews; 2 documents downloaded

**MHRA products, Medicines & Healthcare products Regulatory Agency** <https://products.mhra.gov.uk/>

nusinersen 5 results (SPCs for nusinersen, risdiplam & onasemnogene; 2 x PARs for risdiplam (that appear to be identical)

risdiplam 5 results, as above.

Total: 1 drug review; 1 document downloaded (risdiplam PAR)

**European Medicines Agency (EMA)** <https://www.ema.europa.eu/en/homepage>

*search box on homepage*

nusinersen 3 results, of which 1 relevant *downloaded 2 EPAR reports*

risdiplam 3 results, of which 1 relevant *downloaded 2 EPAR reports*

Total: 2 drug reviews; 4 documents downloaded

**Total:**

**21 technology assessments, reviews or other projects identified**

**31 documents downloaded**

**1 webpage bookmarked (ongoing project)**

Search strategies: update search

**Embase (Ovid)**

Date searched: 10/02/25

Embase <1974 to 2025 February 07>

1 exp hereditary spinal muscular atrophy/ or spinal muscular atrophy/ 15343

2 (spinal muscul* atroph* or SMA).kf,tw. 54358

3 (Werdnig adj Hoffman*).kf,tw. 419

4 (Kugelberg adj Welander*).kf,tw. 216

5 1 or 2 or 3 or 4 [disease terms] 59777

6 nusinersen/ 2228

7 (nusinersen or spinraza* or isis smn* or isis 396443).kf,tn,tw. 1954

8 risdiplam/ 769

9 (risdiplam or evrysdi* or RG 7916 or RG7916).kf,tn,tw. 647

10 onasemnogene abeparvovec/ 1151

11 (onasemnogene or zolgensma* or AVXS-101).kf,tn,tw. 1036

12 6 or 7 or 8 or 9 or 10 or 11 3321

13 Economics/ 243410

14 Cost/ 64449

15 exp Health Economics/ 1102946

16 Budget/ 35724

17 budget*.ti,ab,kw. 51249

18 (economic* or cost or costs or costly or costing or price or prices or pricing or pharmacoeconomic* or pharmaco-economic* or expenditure or expenditures or expense or expenses or financial or finance or finances or financed).ti,kw. 337090

19 (economic* or cost or costs or costly or costing or price or prices or pricing or pharmacoeconomic* or pharmaco-economic* or expenditure or expenditures or expense or expenses or financial or finance or finances or financed).ab. /freq=2 593363

20 (cost* adj2 (effective* or utilit* or benefit* or minimi* or analy* or outcome or outcomes)).ab,kw. 318168

21 (value adj2 (money or monetary)).ti,ab,kw. 4438

22 Statistical Model/ 178408

23 economic model*.ab,kw. 6889

24 Probability/ 162467

25 markov.ti,ab,kw. 40111

26 monte carlo method/ 55765

27 monte carlo.ti,ab,kw. 66961

28 Decision Theory/ 1885

29 Decision Tree/ 26856

30 (decision* adj2 (tree* or analy* or model*)).ti,ab,kw. 61294

31 or/13-30 [Economic Evaluations & Models - Embase. In: CADTH Search Filters Database. Ottawa: CADTH; 2023: https://searchfilters.cadth.ca/link/15] 2159477

32 socioeconomics/ 171156

33 exp Quality of Life/ 734809

34 quality of life.ti,kw. 190195

35 ((instrument or instruments) adj3 quality of life).ab. 5716

36 Quality-Adjusted Life Year/ 39277

37 quality adjusted life.ti,ab,kw. 29278

38 (qaly* or qald* or qale* or qtime* or life year or life years).ti,ab,kw. 50139

39 disability adjusted life.ti,ab,kw. 8202

40 daly*.ti,ab,kw. 7977

41 (sf36 or sf 36 or short form 36 or shortform 36 or short form36 or shortform36 or sf thirtysix or sfthirtysix or sfthirty six or sf thirty six or shortform thirtysix or shortform thirty six or short form thirtysix or short form thirty six).ti,ab,kw. 53057

42 (sf6 or sf 6 or short form 6 or shortform 6 or sf six or sfsix or shortform six or short form six or shortform6 or short form6).ti,ab,kw. 3211

43 (sf8 or sf 8 or sf eight or sfeight or shortform 8 or shortform 8 or shortform8 or short form8 or shortform eight or short form eight).ti,ab,kw. 1099

44 (sf12 or sf 12 or short form 12 or shortform 12 or short form12 or shortform12 or sf twelve or sftwelve or shortform twelve or short form twelve).ti,ab,kw. 13279

45 (sf16 or sf 16 or short form 16 or shortform 16 or short form16 or shortform16 or sf sixteen or sfsixteen or shortform sixteen or short form sixteen).ti,ab,kw. 75

46 (sf20 or sf 20 or short form 20 or shortform 20 or short form20 or shortform20 or sf twenty or sftwenty or shortform twenty or short form twenty).ti,ab,kw. 558

47 (hql or hqol or h qol or hrqol or hr qol).ti,ab,kw. 43557

48 (hye or hyes).ti,ab,kw. 203

49 (health* adj2 year* adj2 equivalent*).ti,ab,kw. 53

50 (pqol or qls).ti,ab,kw. 792

51 (quality of wellbeing or quality of well being or index of wellbeing or index of well being or qwb).ti,ab,kw. 630

52 nottingham health profile*.ti,ab,kw. 1705

53 nottingham health profile/ 700

54 sickness impact profile.ti,ab,kw. 1292

55 sickness impact profile/ 2352

56 health status indicator/ 3602

57 (health adj3 (utilit* or status)).ti,ab,kw. 129489

58 (utilit* adj3 (valu* or measur* or health or life or estimat* or elicit* or disease or score* or weight)).ti,ab,kw. 28203

59 (preference* adj3 (valu* or measur* or health or life or estimat* or elicit* or disease or score* or instrument or instruments)).ti,ab,kw. 21138

60 disutilit*.ti,ab,kw. 1428

61 rosser.ti,ab,kw. 145

62 willingness to pay.ti,ab,kw. 14952

63 standard gamble*.ti,ab,kw. 1247

64 (time trade off or time tradeoff).ti,ab,kw. 2543

65 tto.ti,ab,kw. 2457

66 (hui or hui1 or hui2 or hui3).ti,ab,kw. 3481

67 (eq or euroqol or euro qol or eq5d or eq 5d or euroqual or euro qual).ti,ab,kw. 43357

68 duke health profile.ti,ab,kw. 124

69 functional status questionnaire.ti,ab,kw. 182

70 dartmouth coop functional health assessment*.ti,ab,kw. 14

71 or/32-70 [ Economic - Health Utilities / Quality of Life - Standard - Embase. In: CADTH Search Filters Database. Ottawa: CADTH; 2022: https://searchfilters.cadth.ca/link/18.] 1101316

72 5 and 12 and 31 455

73 5 and 12 and 71 287

74 72 or 73 613

**MEDLINE (Ovid)**

Date searched: 10/02/25

Ovid MEDLINE(R) ALL <1946 to February 07, 2025>

1 exp Muscular Atrophy, Spinal/ 6996

2 (spinal muscul* atroph* or SMA).mp. 35853

3 (Werdnig adj Hoffman*).mp. 404

4 (Kugelberg adj Welander*).mp. 214

5 1 or 2 or 3 or 4 [disease terms] 37730

6 (nusinersen or spinraza* or isis smn* or isis 396443).mp. 945

7 (risdiplam or evrysdi* or RG 7916 or RG7916).mp. 246

8 (onasemnogene or zolgensma* or AVXS-101).mp. 357

9 6 or 7 or 8 [intervention or comparator terms] 1196

10 Economics/ 27545

11 exp "Costs and Cost Analysis"/ 276364

12 Economics, Nursing/ 4013

13 Economics, Medical/ 9299

14 Economics, Pharmaceutical/ 3156

15 exp Economics, Hospital/ 26108

16 Economics, Dental/ 1922

17 exp "Fees and Charges"/ 31617

18 exp Budgets/ 14316

19 budget*.ti,ab,kf. 39660

20 (economic* or cost or costs or costly or costing or price or prices or pricing or pharmacoeconomic* or pharmaco-economic* or expenditure or expenditures or expense or expenses or financial or finance or finances or financed).ti,kf. 308607

21 (economic* or cost or costs or costly or costing or price or prices or pricing or pharmacoeconomic* or pharmaco-economic* or expenditure or expenditures or expense or expenses or financial or finance or finances or financed).ab. /freq=2 430592

22 (cost* adj2 (effective* or utilit* or benefit* or minimi* or analy* or outcome or outcomes)).ab,kf. 241315

23 (value adj2 (money or monetary)).ti,ab,kf. 3381

24 exp models, economic/ 16724

25 economic model*.ab,kf. 4671

26 markov chains/ 16819

27 markov.ti,ab,kf. 32329

28 monte carlo method/ 33790

29 monte carlo.ti,ab,kf. 65797

30 exp Decision Theory/ 14080

31 (decision* adj2 (tree* or analy* or model*)).ti,ab,kf. 47772

32 or/10-31 [Economic Evaluations & Models - MEDLINE. In: CADTH Search Filters Database. Ottawa: CADTH; 2024: https://searchfilters.cadth.ca/link/16] 993736

33 "Value of Life"/ 5834

34 Quality of Life/ 300447

35 quality of life.ti,kf. 132958

36 ((instrument or instruments) adj3 quality of life).ab. 4160

37 Quality-Adjusted Life Years/ 17424

38 quality adjusted life.ti,ab,kf. 19723

39 (qaly* or qald* or qale* or qtime* or life year or life years).ti,ab,kf. 32702

40 Disability-Adjusted Life Years/ 782

41 disability adjusted life.ti,ab,kf. 7103

42 Healthy Life Expectancy/ 78

43 (daly* or disability free life expectanc* or haly* or health* life expectanc*).ti,ab,kf. 8273

44 (sf36 or sf 36 or short form 36 or shortform 36 or short form36 or shortform36 or sf thirtysix or sfthirtysix or sfthirty six or sf thirty six or shortform thirtysix or shortform thirty six or short form thirtysix or short form thirty six).ti,ab,kf. 33083

45 (sf6 or sf 6 or short form 6 or shortform 6 or sf six or sfsix or shortform six or short form six or shortform6 or short form6).ti,ab,kf. 2919

46 (sf8 or sf 8 or sf eight or sfeight or shortform 8 or shortform 8 or shortform8 or short form8 or shortform eight or short form eight).ti,ab,kf. 673

47 (sf12 or sf 12 or short form 12 or shortform 12 or short form12 or shortform12 or sf twelve or sftwelve or shortform twelve or short form twelve).ti,ab,kf. 8564

48 (sf16 or sf 16 or short form 16 or shortform 16 or short form16 or shortform16 or sf sixteen or sfsixteen or shortform sixteen or short form sixteen).ti,ab,kf. 43

49 (sf20 or sf 20 or short form 20 or shortform 20 or short form20 or shortform20 or sf twenty or sftwenty or shortform twenty or short form twenty).ti,ab,kf. 478

50 (hql or hqol or h qol or hrqol or hr qol).ti,ab,kf. 27670

51 (hye or hyes).ti,ab,kf. 79

52 (health* adj2 year* adj2 equivalent*).ti,ab,kf. 48

53 (pqol or qls).ti,ab,kf. 492

54 (quality of wellbeing or quality of well being or index of wellbeing or index of well being or qwb).ti,ab,kf. 524

55 nottingham health profile*.ti,ab,kf. 1281

56 sickness impact profile.ti,ab,kf. 1106

57 exp health status indicators/ 356349

58 (health adj3 (utilit* or status)).ti,ab,kf. 102692

59 (utilit* adj3 (valu* or measur* or health or life or estimat* or elicit* or disease or score* or weight)).ti,ab,kf. 17982

60 (preference* adj3 (valu* or measur* or health or life or estimat* or elicit* or disease or score* or instrument or instruments)).ti,ab,kf. 16278

61 disutilit*.ti,ab,kf. 715

62 rosser.ti,ab,kf. 113

63 willingness to pay.ti,ab,kf. 10202

64 standard gamble*.ti,ab,kf. 940

65 (time trade off or time tradeoff).ti,ab,kf. 1768

66 tto.ti,ab,kf. 1591

67 (hui or hui1 or hui2 or hui3).ti,ab,kf. 2183

68 (eq or euroqol or euro qol or eq5d or eq 5d or euroqual or euro qual).ti,ab,kf. 26494

69 duke health profile.ti,ab,kf. 96

70 functional status questionnaire.ti,ab,kf. 136

71 dartmouth coop functional health assessment*.ti,ab,kf. 14

72 or/33-71 [Economic - Health Utilities / Quality of Life - MEDLINE. In: CADTH Search Filters Database. Ottawa: CADTH; 2024: https://searchfilters.cadth.ca/link/19. Accessed 2024-01-26.] 822305

73 5 and 9 and 32 88

74 5 and 9 and 72 80

75 73 or 74 141

**Science Citation Index and Conference Proceedings (Web of Science)**

Date searched: 10/02/25

Database: Web of Science Core Collection

Editions searched/Entitlements: WOS.SCI: 1900 to 2025, WOS.ISTP: 1990 to 2025

| # | Search Query | Results |
| --- | --- | --- |
| 1 | TS=(nusinersen OR spinraza* OR "isis smn*" OR "isis 396443") | 1450 |
| 2 | TS=(risdiplam OR evrysdi* OR "RG 7916" OR RG7916) | 358 |
| 3 | TS=(onasemnogene or zolgensma* or "AVXS-101") | 547 |
| 4 | #1 OR #2 OR #3 | 1970 |
| 5 | TS=(("spinal muscul*" NEAR/0 atroph*) OR SMA) | 53955 |
| 6 | TS=(Werdnig NEAR/0 Hoffman*) | 513 |
| 7 | TS=(Kugelberg NEAR/0 Welander*) | 168 |
| 8 | #5 OR #6 OR #7 | 54221 |
| 9 | TS=(cost* or economic* or pharmacoeconomic* or pharmaco-economic* or price or prices or pricing or expenditure* or expense* or financial or finance or finances or financed or budget* or (value NEAR/1 (money OR monetary)) or (economic NEAR/1 model*) or markov or monte carlo or (decision NEAR/1 (tree* or analy* or model*))) | 4041452 |
| 10 | #4 AND #8 AND #9 | 135 |
| 11 | TS=("value of life" or "quality of life" or qol or hrql or hrqol or (("quality adjusted life" OR "disability adjusted life") NEAR/0 year*) or qaly* or qald* or qale* or qtime* or "life year" or "life years" or daly* or ("disability free life" NEAR/0 expectanc*) or haly* or (health* NEAR/0 ("life expectance" or "life expectancy") or icer or "euro-qol" or euroqual or "euro qual" or utilit* or disutilit* or (net NEAR/0 benefit*) or (contingent NEAR/0 valuation*) or (preference* NEAR/2 (valu* or measur* or health or life or estimat* or elicit* or disease or score* or instrument or instruments)) or euroqol or "euro qol" or eq5d or eq-5d or "short-form 36" or "shortform 36" or sf-36 or sf36 or sf-6d or sf6d or sf-12 or sf12 or "health utilities index" or hui or hui1 or hui2 or hui3 or (time NEAR/0 trade*) or tto or "standard gamble" or sg or markov or (decision NEAR/1 model*) or (visual NEAR/0 analog*) or "discrete choice" or ((health* NEAR/0 year*) NEAR/0 equivalen*) or (health NEAR/0 stat*) or (willing* NEAR/1 pay) or resource* or wellbeing or well-being)) | 2756518 |
| 12 | #4 AND #8 AND #11 | 155 |
| 13 | #10 OR #12 Editions: WOS.SCI,WOS.ISTP | 238 |

**International HTA database (INAHTA)** <https://database.inahta.org/>

Date searched: 10/02/25

(("Muscular Atrophy, Spinal"[mhe]) OR ("spinal muscular atrophy" OR "spinal muscular atrophies" OR SMA OR "Werdnig Hoffman" OR "Kugelberg Welander"))

41 results

**CEA Registry (Tufts Medical Center)** <https://cear.tuftsmedicalcenter.org/>

Date searched: 10/02/25

Advanced search screen, Methods:

keyword:("spinal" AND "muscular" AND "atrophy") OR keyword:"SMA" 15 results, of which 2 duplicates

keyword:"nusinersen" OR keyword:"spinraza" OR keyword:"risdiplam" OR keyword:"evrysdi" OR keyword:"onasemnogene" OR keyword:"zolgensma" 4 results, of which 3 already found above

Total unique results: 14

**EconPapers (RePEc)** <https://econpapers.repec.org/scripts/search.pf>

Date searched: 10/02/25

Advanced search screen; Free text search box

nusinersen OR spinraza OR risdiplam OR evrysdi OR onasemnogene OR zolgensma 15 results

**Internet (Google)** <https://www.google.co.uk/>

Date searched: 11/02/25

*Records were retrieved (added to EndNote library) only if potentially relevant to the review, and not already found via the database/other searches*

economic evaluation nusinersen OR risdiplam OR onasemnogene OR spinraza OR evrysdi OR zolgensma 1 Jan 2024 – Today browsed first 20 results; 1 record retrieved.

cost effectiveness nusinersen OR risdiplam OR onasemnogene OR spinraza OR evrysdi OR zolgensma 1 Jan 2024 – Today browsed first 20 results; 0 records retrieved.

health technology assessment nusinersen OR risdiplam OR onasemnogene OR spinraza OR evrysdi OR zolgensma 1 Jan 2024 – Today browsed first 20 results; 0 records retrieved.

Total: 1 record retrieved.

**Websites**

Date searched: 11/02/25

**National Institute for Health and Care Excellence (NICE)** <https://www.nice.org.uk/guidance/published?sp=on>

*Search for published guidance*

*Filter: Type: Guidance*

*Filter: Last updated date: From 01/01/2024 To 11/02/2025*

Filter by keyword or reference number:

Spinal muscular atrophy 0 results

SMA 0 results

nusinersen 0 results

risdiplam 0 results

onasemnogene 0 results

Total: 0 new or updated results since original search

**Scottish Medicines Consortium (SMC)** <https://www.scottishmedicines.org.uk/>

*Search box on homepage*

spinal muscular atrophy 6 results, 0 new/updated since 2024

nusinersen 2 results, 0 new

risdiplam 2 results, 0 new

onasemnogene 2 results, 0 new

Total: 0 new or updated results since original search

**Canada’s Drug Agency (CDA-AMC)** <https://www.cda-amc.ca/find-reports>

*‘View all reports’ selected; ‘Last updated’ 01/01/2024 to 11/02/2025*

Spinal muscular atrophy 0 new results

SMA 22 results, of which 0 relevant

nusinersen 0 new results

risdiplam 0 new results

onasemnogene 0 new results

Checked ongoing project webpage identified previously: <https://www.cadth.ca/health-technology-assessment-recommendations-and-managed-entry-agreements-related-optimizing> (now redirects to: <https://www.cda-amc.ca/health-technology-assessment-recommendations-and-managed-entry-agreements-related-optimizing> ) *Project Status remains “in progress”*

Total: 0 new or updated results since original search

**Institute for Clinical and Economic Review (ICER)** <https://icer.org/explore-our-research/assessments/>

*search by keyword; no filters applied*

spinal muscular atrophy 2 results, 1 new but not relevant (about apitegromab)

SMA 10 results, of which 2 new but not relevant

nusinersen 1 result, 0 new

risdiplam 0 results

onasemnogene 1 result, 0 new

Total: 0 new or updated results since original search

**Drugs@FDA , U.S. Food & Drug Administration** <https://www.accessdata.fda.gov/scripts/cder/daf/>

nusinersen 0 new review documents since previous search

risdiplam 0 new review documents since previous search

onasemnogene 0 results

zolgensma 0 results

Total: 0 new or updated review documents since original search

**MHRA products, Medicines & Healthcare products Regulatory Agency** <https://products.mhra.gov.uk/>

nusinersen 7 results, 2 new but not relevant (SPCs or patient information leaflets)

risdiplam 7 results, as above

onasemnogene 7 results, as above

Total: 0 new or updated review documents since original search

**European Medicines Agency (EMA)** <https://www.ema.europa.eu/en/homepage>

*‘Find medicine’ search box on homepage*

nusinersen 1 result; EPAR has been updated in 2024 but only with minor administrative changes/changes to SmPC, see <https://www.ema.europa.eu/en/documents/procedural-steps-after/spinraza-epar-procedural-steps-taken-scientific-information-after-authorisation_en.pdf>

*0 downloaded*

risdiplam 1 result; 2 EPAR Assessment Report Variations in 2024, but no economic/cost-effectiveness data

Total: 0 documents downloaded

**Appendix 3: Example of a complete data extraction sheet**

Furter completed sheets are available on request.

**Date: 04/03/2024**

**Study ID: -**

**Name of first reviewer: MY**

**Name of second reviewer: PA**

Table 3: Completed data extraction sheet of an economic analysis

| Study details | |
| --- | --- |
| Study title | Pharmacoeconomic Review Report(Resubmission): Nusinersen (Spinraza): (Biogen Canada Inc): Indication: Treatment of patients with 5q SMA |
| First author | Canadian Agency for Drugs and Technologies in Health (CADTH) |
| Co-authors | - |
| Source of publication  Journal yy;vol(issue):pp | Version: Final (With Redactions)  Publication Date: April 2019  Report Length: 35 Pages |
| Publication link | <https://www.cadth.ca/sites/default/files/cdr/pharmacoeconomic/sr0576-spinraza-resubmission-pharmacoeconomic-report.pdf> |
| Language | English |
| Publication type | HTA Report |
| Inclusion criteria/study eligibility/PICOS | |
| Population | Patients with SMA (type 1, 2 and 3) |
| Intervention(s) | Nusinersen (Spinraza) |
| Comparator(s) | standard of care (real-world care) |
| Outcome(s) | Life-years (LYs), and quality-adjusted life-years (QALYs) |
| Study design | Economic Evaluation (cost-utility analyses) |
| Methods | |
| Target population and subgroups | Patients with 5q SMA — stratified by SMA type — type I, II, and III |
| Setting and location | Canada |
| Approach to engagement with patients and others affected by the study | It is not clear |
| Study perspective | Canadian public health care system |
| Comparators | Standard of care (or real-world care, which includes supportive symptomatic treatment of respiratory, nutritional, and orthopaedic function decline) for patients with 5q SMA. |
| Time horizon | Time horizon:   - SMA type 1: 25 years - SMA type 2: 50 years - SMA type 3: 80 years   Cycle length:   - SMA type 1: patients could transition between health states at 2, 6, 10, 13, and 14 months. Subsequent cycles were every four months, which conformed to the timing of dosages of Nusinersen. - SMA type 2: For the first 15 months of the model, the cycle length was three months conforming to the timing of clinical assessment in the CHERISH study. Subsequent cycles were every four months. - SMA type 3: 3 months (for the first 27 months, subsequent cycles every 4 months) |
| Discount rate | 1.5% costs and benefits per annum |
| Outcome(s) | Life-years (LYs), and quality-adjusted life-years (QALYs) |
| Measurement of effectiveness | Philadelphia Infant Test of Neuromuscular Disorders (CHOP INTEND) scores  Hammersmith Functional Motor Scale – Expanded (HFMSE) scores |
| Measurement and valuation of preference-based outcomes | Patient: Types 1 and 3 SMA: unpublished utility value analyses by five experts in SMA  Type 2 SMA: QoL data from CHERISH mapped to EQ-5D  For long-term survival, data were used that were derived from a survival analysis of observational data from Zerres and Rudnik-Schoneborn. |
| Methods for identifying resource use | According to Klug et al. study  Focus groups with input from clinicians, health-economists and patient representatives |
| Resource use and costs | According to Klug et al. study  direct medical COI (Outpatient medical costs, Inpatient medical costs, Rehabilitation costs (in-/outpatient), Drug treatment costs, Costs for use of rehabilitation services, Costs for artificial nutrition, Costs for medical aids, Costs for respiratory management)  direct non-medical COI (Costs for housing, Costs for personal assistance for school and work attendance, Travel expenses, Informal care costs, Costs for legal advice, Costs for constructional modifications to house, Costs for constructional modifications to automobile, Other expenditures) |
| Data source of resource use | Health costs appear to be derived from a study of SMA costs in Germany |
| Currency, price date and conversion | All cost reported in Canadian dollars- Costs (currency, year): CAD, 2017 |
| Analytic approach and model type (if applicable) | Model:   - Three distinct Markov models were developed for three SMA types: type I, type II, and type III   States:   - In the SMA type I model, health states included baseline clinical status; whether clinical status improved, worsened, or had no improvement; milestones consistent with SMA type II (e.g., sits without support, stands with assistance, walks with assistance, and stand/walks unaided); and death. - In the SMA type II model, health states included baseline clinical status; whether clinical status worsened, had no improvement, had mild improvement, or had moderate improvement; whether the patient could stand or walk with assistance and milestones consistent with SMA type III (e.g., stand unaided and walks unaided); and death. - In the SMA type III model, health states included non-ambulatory, ambulatory, and death.   Other points:   - expected values of costs, QALYs, and Lys were obtained through probabilistic analysis.   Transition probabilities:   - For SMA type I, the transition probabilities for Nusinersen and real-world care (RWC) were obtained from the ENDEAR trial. - For SMA type II, the transition probabilities for Nusinersen and RWC were obtained from the CHERISH trial. - For SMA type III, Transition probabilities during the study period (first 24 months) between non-ambulatory and ambulatory for patients receiving Nusinersen were obtained from the CS2 and CS12 studies. [CONFIDENTIAL manufacturer's submission] |
| Assumptions | For treatment discontinuation, it was assumed that individuals would stop treatment after scoliosis surgery or after entering the worsening state.  It was assumed that patients receiving Nusinersen would have a reduced risk of mortality up to 50 months beyond the trial follow-up period. In addition, it was assumed that all patients who reached milestones consistent with SMA type II would experience mortality rates associated with SMA type II.  Long-term mortality was assumed to be the same as for the general population. |
| Results | |
| Study parameters | Disease progression during study period, Disease progression after study period, Mortality during study period, Mortality post study period, Utility values, Cost data |
| Total costs and outcomes  (Manufacturer’s Base Case) | \| Total QALYs \| Total LYs \| Total costs \| \| --- \| --- \| --- \| \| SMA type 1:  o Nusinersen: 3.919  o RWC: -0.881  SMA type 2:  o Nusinersen: 23.278  o RWC: 19.602  SMA type 3:  o Nusinersen: 12.053  o RWC: 10.490 \| SMA type 1:  o Nusinersen: 8.373  o RWC: 3.583  SMA type 2:  o Nusinersen: 28.527  o RWC: 26.348  SMA type 3:  o Nusinersen: 44.155  o RWC: 44.155 \| SMA type 1:  o Nusinersen: $3,534,854  o RWC: $339,683  SMA type 2:  o Nusinersen: $8,336.271  o RWC: $708,620  SMA type 3:  o Nusinersen: $5,554,707  o RWC: $1,091,307 \| |
| Characterising uncertainty | - Probabilistic sensitivity analysis - Some scenarios about Alternative Utility Values, Alternative Progression Assumptions, Alternative Survival Assumptions and Additional Price Analyses. |
| Discussion | |
| Study findings  (Manufacturer’s Base Case) | ICER/QALY:   - SMA type 1: Nusinersen vs RWC: $665,570 - SMA type 2: Nusinersen vs RWC: $2,075,435 - SMA type 3: Nusinersen vs RWC: $2,855,818   The manufacturer reported that the probability that Nusinersen was cost-effective assuming a willingness-to-pay threshold of $300,000 per QALY was 0% for all SMA types. The manufacturer reported a number of scenario analyses; however, for all SMA types, the incremental cost per QALY gained for Nusinersen exceeded $500,000 in all analyses. |
| Limitations | 1. Utility values were derived from unpublished studies provided for Biogen Idec, which the CADTH Common Drug Review (CDR) did not consider had appropriate methodology for the estimation of utility. 2. The manufacturer made inappropriate assumptions relating to disease progression for patients with SMA types I, II, and III receiving Nusinersen. 3. The manufacturer made inappropriate assumptions relating to mortality within SMA types I and II. 4. Certain health states within the model were inappropriate as they were reflective relative rather than absolute health states. 5. The manufacturer’s submission did not allow further stratification by disease status within SMA type, which would have been highly informative. 6. The CDR clinical expert has raised a number of concerns with the clinical trial data for Nusinersen, which undermines the ability to facilitate the economic evaluation. This particularly relates to the lack of appropriate clinical data for assessing the effectiveness of Nusinersen in SMA type III. 7. The manufacturer did not provide new economic information as part of their resubmission and did not further address the previously cited limitations. |
| Generalisability | 1. Limited Population Representation in Clinical Trials: The lack of representation of the entire spectrum of patients with SMA in the clinical trials, especially for SMA type III, raises concerns about the generalizability of the study findings to real-world clinical practice. The subset of patients included in the trials may not fully reflect the diverse population that would receive Nusinersen. 2. Inadequate Comparative Clinical Trial Data: The absence of comprehensive comparative clinical trial data for SMA type III limits the ability to generalize the effectiveness of Nusinersen across all subtypes of 5q SMA. The relevance and applicability of the study conclusions may be compromised due to this lack of specific data for certain patient groups. 3. Potential Age-Related Treatment Effectiveness Variability: Subgroup analysis indicating varying effectiveness of Nusinersen based on age categories suggests the importance of considering age-related factors in evaluating treatment outcomes. Generalizing the cost-effectiveness of Nusinersen without stratifying by age may overlook significant variations in treatment response within different age groups. |
| Other | |
| Source of funding | Canada’s federal, provincial, and territorial governments |
| Conflicts of interest | The majority of information came from the company submission. There are some possibilities regarding conflicts of interest. |
| Comments | 1. Need for Absolute Health States: The economic model's reliance on relative states linked to patients' baseline status creates ambiguity in assessing actual functional improvements. Shifting towards absolute states representing current functioning levels could enhance the model's accuracy and relevance. 2. Questionable Utility Value Sources: The utilization of unpublished analyses and mapping exercises to derive utility values for different SMA types raises concerns about data transparency and validity. Clear and specific sources are crucial to ensuring the reliability of the economic evaluation. 3. Biased Assumptions in Disease Progression and Mortality: Biased assumptions favoring Nusinersen in disease progression post-clinical studies and mortality based on reached milestones undermine the objectivity of the economic model. Unfounded biases can skew cost-effectiveness outcomes and should be rectified. 4. Inadequate Representation of Population in Clinical Trials: Critically evaluating the lack of representation of the entire SMA population in clinical trials, particularly for SMA type III, is essential. Diverse patient demographics must be considered to obtain a comprehensive understanding of Nusinersen's effectiveness in real-world scenarios. 5. Importance of Stratified Analysis by Age and Disease Status: Conducting further stratified analyses based on age and disease status, as indicated by subgroup analysis findings, can provide valuable insights into Nusinersen's effectiveness across different patient groups. Enhancing stratification can lead to more informative and targeted cost-effectiveness assessments. |
| Authors conclusion | |
| In alignment with the manufacturer’s results of its pharmacoeconomic submission, CDR found that Nusinersen was not a cost-effective treatment for patients with 5q SMA types I, II, or III.  This finding has not been affected by the clinical information provided within the manufacturer’s resubmission. | |
| Reviewer’s conclusion | |
| The limitations in the economic model, including relative states and questionable utility value sources, along with biases in disease progression assumptions, cast doubt on the cost-effectiveness evaluation of Nusinersen for 5q SMA. Concerns raised by clinical experts about trial data discrepancies highlight the need for further stratified analysis and data refinement. Despite reanalysis aligning with non-cost-effectiveness, substantial uncertainties remain, particularly in assessing SMA type III. Given these challenges, Nusinersen's cost-effectiveness across SMA types I, II, and III is dubious. Continued research and data accuracy improvements are vital for reliable pharmacoeconomic assessments in 5q SMA treatment. | |

**Appendix 4: Reporting quality assessment**

Table 4 and Table 5 report the reporting quality of studies against the CHEERS II statement.[5]

Table 4: Critical appraisal using the CHEERS checklist (Part I)

| **CHEERS criteria** | **Study and location where item is reported (page for location refer to PDF page)** | | | | | | | | | |
| --- | --- | --- | --- | --- | --- | --- | --- | --- | --- | --- |
|  | **NICE-Nusinersen TA588** | **NICE-Risdiplam TA755** | **NICE-Onasemnogene HST15-type1** | **NICE-Onasemnogene HST24-pre-symptomatic** | **ICER Spinraza and zolgensma 2019** | **CADTH Nusinersen-2019** | **CADTH Risdiplam**  **2021** | **CADTH Onasemnogene -2021** | **Meijer**  **2023** | **Broekhoff- 2021** |
| ***Title*** | | | | | | | | | | |
| Identify the study as an economic evaluation and specify the interventions being compared | Yes | Yes | Yes | Yes | Yes  P:1 | Yes  P:1 | Yes  P:1 | Yes  P:1 | Yes  P:1 | Yes  P:1 |
| ***Abstract*** | | | | | | | | | | |
| Provide a structured summary that highlights context, key methods, results, and alternative analyses. | No | No | No | No | Yes  P:8 | Yes  P:6-7 | Yes  P:168-169 | Yes  P:7-9 | Yes  P:3 | Yes  P:1 |
| ***Introduction*** | | | | | | | | | | |
| Give the context for the study, the study question, and its practical  relevance for decision making in policy or practice. | No | Yes | Yes | Yes | Yes  P:49-61 | Yes  P:8-10 | Yes  P:25-30, 44-47 | No | Yes  P:8-13 | Yes  P:1-2 |
| ***Methods*** | | | | | | | | | | |
| Health economic analysis plan: Indicate whether a health economic analysis plan was developed and where available. | No | No | No | No | No | No | No | No | No | No |
| Study population: Describe characteristics of the study population (such as age range, demographics, socioeconomic, or clinical characteristics). | Yes | Yes | Yes | Yes | Yes  P:101 | Yes  P:12 | Yes  P:168 | Yes  P:11 | Yes  P:14 | Yes  P:4 |
| Setting and Location: Provide relevant contextual information that may inﬂuence ﬁndings. | Yes | Yes | Yes | Yes | Yes  P:57 | Yes  P:2 | Yes  P:2 | Yes  P:2 | Yes  P:14 | Yes  P:4 |
| Comparators: Describe the interventions or strategies being compared and why chosen. | Yes | Yes | Yes | Yes | Yes  P:55 | Yes  P:9 | Yes  P:168 | Yes  P:7 | Yes  P:14 | Yes  P:4 |
| Perspective: State the perspective(s) adopted by the study and why chosen. | Yes | Yes | Yes | Yes | Yes  P:27 | Yes  P:12 | Yes  P:172 | Yes  P:7 | Yes  P:14 | Yes  P:4 |
| Time Horizon: State the time horizon for the study and why appropriate. | Yes | Yes | Yes | Yes | Yes  P:29 | Yes  P:12 | Yes  P:168 | Yes  P:7 | Yes  P:15 | Yes  P:4 |
| Discount Rate: Report the discount rate(s) and reason chosen. | Yes | Yes | Yes | Yes | Yes  P:27 | Yes  P:12 | Yes  P:172 | Yes  P:11 | Yes  P:15 | Yes  P:4 |
| Selection of outcomes: Describe what outcomes were used as the measure(s) of beneﬁt(s) and harm(s). | Yes | Yes | Yes | Yes | Yes  P:56 | Yes  P:12 | Yes  P:168 | Yes  P:7 | Yes  P:15 | Yes  P:4-5 |
| Measurement of outcomes: Describe how outcomes used to capture beneﬁt(s) and harm(s) were measured. | Yes | Yes | Yes | Yes | Yes  P:11 | Yes  P:15-16 | Yes  P:174-175 | Yes  P:13 | Yes  P:22-23 | No |
| Valuation of outcomes: Describe the population and methods used to measure and value outcomes. | Yes | Yes | Yes | Yes | Yes  P: 11 | Yes  P:15-16 | Yes  P:174 | Yes  P:13 | Yes  P:22-23 | Yes  P:4-5 |
| Measurement and valuation of resources and costs: Describe how costs were valued. | Yes | Yes | Yes | Yes | Yes  P: 111-115 | Yes  P:13 | Yes  P:174-175 | Yes  P:13 | Yes  P:23-25 | Yes  P:5 |
| Currency, price date, and conversion: Report the dates of the estimated resource quantities and unit costs, plus the currency and year of conversion. | Yes | No | Yes | Yes | Yes  P:11-115 | Yes  P:14 | No | No | Yes  P:23 | Yes  P:5 |
| Rationale and description of model: If modelling is used, describe in detail, and why used. Report if the model is publicly available and where it can be accessed. | yes | yes | Yes | Yes | Yes  P:99-101 | No | Yes  P:171 | Yes  P:11 | Yes  P:15-16 | No |
| Analytics and assumptions: Describe any methods for analysing or statistically transforming data, any extrapolation methods, and approaches for validating any model used. | Yes | Yes | Yes | Yes | Yes  P:102-103 | No | Yes  P:171-174 | Yes  P:20 | Yes  P:36-37 | Yes  P:5 |
| Characterizing heterogeneity: Describe any methods used for estimating how the results of the study vary for subgroups. | Yes | Yes | Yes | Yes | Yes  P:106-109 | Yes  30-31 | Yes  P:171 | Yes  P:11 | Yes  P:12-13 | No |
| Characterizing distributional effects: Describe how impacts are distributed across different individuals or adjustments made to reﬂect priority populations. | Yes | Yes | Yes | Yes | Yes  P:106-109 | Yes  P:14 | Yes  P:171 | No | No | No |
| Characterizing uncertainty: Describe methods to characterise any sources of uncertainty in the analysis. | Yes | Yes | Yes | Yes | Yes  P:115 | No | Yes  P:176-181 | Yes  P:14 | Yes  P:24-26 | Yes  P:5-6 |
| Approach to engagement with patients and others affected by the study: Describe any approaches to engage patients or service recipients, the general public, communities, or stakeholders (such as clinicians or payers) in the design of the study. | Yes | Yes | Yes | Yes | Yes  P:31 | No | Yes  P:30-34 | Yes  P:10 | No | No |
| ***Results*** | | | | | | | | | | |
| Study parameters: Report all analytic inputs (such as values, ranges, references) including uncertainty or distributional assumptions. | Yes | Yes | Yes | Yes | Yes  P: 117-136 | No | No | Yes  P:38 | Yes  P:25 | Yes  P:7 |
| Summary of main results: Report the mean values for the main categories of costs and outcomes of interest and summarise them in the most appropriate overall measure. | Yes | No | Yes | No | Yes  P:141-143 | Yes  P:14, 20 | Yes  P:182 | Yes  P:7, 24 | Yes  P:29 | Yes  P:5 |
| Effect of uncertainty: Describe how uncertainty about analytic judgments, inputs, or  projections affect ﬁndings. Report the effect of choice of discount rate and time horizon, if applicable. | Yes | Yes | Yes | Yes | Yes  P: 117-136 | No | Yes  P:176-181 | Yes  P:25-26 | Yes  P:29-32 | Yes  P:6-7 |
| Effect of engagement with patients and others affected by the study: Report on any difference patient/service recipient, general public, community, or stakeholder involvement made to the approach or ﬁndings of the study | Yes | Yes | Yes | Yes | No | No | No | No | No | No |
| ***Discussion*** | | | | | | | | | | |
| Study ﬁndings, limitations, generalizability, and current knowledge: Report key ﬁndings, limitations, ethical or equity considerations not captured, and how these could affect patients, policy, or practice. | Yes | Yes | Yes | Yes | Yes  P:139-141 | Yes  P: 6, 15-19 | Yes  P: 169 | Yes  P:15-19, 26 | Yes  P:35-36 | Yes  P:9-10 |
| ***Other*** | | | | | | | | | | |
| Source of Funding: Describe how the study was funded and any role of the funder in the identiﬁcation, design, conduct, and reporting of the analysis | No | No | No | No | Yes  P:3 | Yes  P: 2 | Yes  P: 2 | Yes  P: 2 | No | Yes  P:10 |
| Conflicts of Interest: Report authors conﬂicts of interest according to journal or International Committee of Medical Journal Editors requirements. | Yes | Yes | Yes | Yes | Yes  P:270-272 | Yes  P: 2 | Yes  P: 2 | Yes  P: 2 | No | Yes  P:10 |

Table 5: Critical appraisal using the CHEERS checklist (Part II)

| **CHEERS criteria** | **Study and location where item is reported (page for location refer to PDF page)** | | | | | | | | | | |
| --- | --- | --- | --- | --- | --- | --- | --- | --- | --- | --- | --- |
|  | **SMC Nusinersen- 2018** | **SMC Onasemnogene -2021** | **SMC Risdiplam-2022** | **Connock et al., 2020** | **Dean et al. 2021** | **Malone et al. 2019** | **Wang et al. 2022** | **Zuluaga‑Sanchez et al. 2019** | **Thokala et al. 2020** | **NCPE-2017** | **Khuntha S, et al., 2025** |
| ***Title*** | | | | | | | | | | | |
| Identify the study as an economic evaluation and specify the interventions being compared | No | No | No | Yes  P:1 | Yes  P:1 | Yes  P:1 | Yes  P:1 | Yes  P:1 | Yes  P:1 | Yes  P:1 | Yes  P:1 |
| ***Abstract*** | | | | | | | | | | | |
| Provide a structured summary that highlights context, key methods, results, and alternative analyses. | No | No | No | Yes  P:2 | Yes  P:1 | Yes  P:1 | Yes  P:1 | Yes  P:1 | Yes  P:1 | No | Yes  P:1 |
| ***Introduction*** | | | | | | | | | | | |
| Give the context for the study, the study question, and its practical  relevance for decision making in policy or practice. | Yes  P:3-9 | Yes  P:2-8 | Yes  P:2-9 | Yes  P:2-3 | Yes  P:1-3 | Yes  P:1-2 | Yes  P:1-3 | Yes  P:1-3 | Yes  P:1-2 | No | Yes  P:1-2 |
| ***Methods*** | | | | | | | | | | | |
| Health economic analysis plan: Indicate whether a health economic analysis plan was developed and where available. | No | No | No | No | No | No | No | No | No | No | No |
| Study population: Describe characteristics of the study population (such as age range, demographics, socioeconomic, or clinical characteristics). | Yes  P:11 | Yes  P:9 | Yes  P:10 | Yes  P:3 | Yes  P:1 | Yes  P:2 | Yes  P:2 | Yes  P:5 | Yes  P:2 | No | Yes  P:3 |
| Setting and Location: Provide relevant contextual information that may inﬂuence ﬁndings. | Yes  P:1 | Yes  P:1 | Yes  P:1 | Yes  P:3 | Yes  P:1 | Yes  P:2 | Yes  P:2 | Yes  P:5 | Yes  P:2 | Yes  P:2 | Yes  P:3 |
| Comparators: Describe the interventions or strategies being compared and why chosen. | Yes  P:11 | Yes  P:9 | Yes  P:10 | Yes  P:3 | Yes  P:3 | Yes  P:2 | Yes  P:3 | Yes  P:5 | Yes  P:2 | Yes  P:6 | Yes  P:3 |
| Perspective: State the perspective(s) adopted by the study and why chosen. | Yes  P:11 | Yes  P:10 | No | Yes  P:3 | Yes  P:1 | Yes  P:2 | Yes  P:3 | Yes  P:5 | Yes  P:2 | Yes  P:5 | Yes  P:3 |
| Time Horizon: State the time horizon for the study and why appropriate. | Yes  P:11 | Yes  P:10 | Yes  P:10 | Yes  P:3 | Yes  P:7 | Yes  P:2 | Yes  P:3 | Yes  P:5 | Yes  P:2 | Yes  P:5 | Yes  P:3 |
| Discount Rate: Report the discount rate(s) and reason chosen. | No | No | No | No | Yes  P:7 | Yes  P:3 | Yes  P:3 | Yes  P:5 | Yes  P:2 | Yes  P:6 | Yes  P:3 |
| Selection of outcomes: Describe what outcomes were used as the measure(s) of beneﬁt(s) and harm(s). | Yes  P:11 | Yes  P:11 | Yes  P:13 | Yes  P:3 | Yes  P:1 | Yes  P:2-3 | Yes  P:6 | Yes  P:5 | Yes  P:2 | Yes  P:1 | Yes  P:4 |
| Measurement of outcomes: Describe how outcomes used to capture beneﬁt(s) and harm(s) were measured. | Yes  P:12 | Yes  P:10 | Yes  P:11 | Yes  P:3 | Yes  P:3 | Yes  P:6-7 | Yes  P:3 | Yes  P:13-14 | Yes  P:5-6 | Yes  P:5 | Yes  P:4 |
| Valuation of outcomes: Describe the population and methods used to measure and value outcomes. | Yes  P:12 | Yes  P:10-11 | Yes  P:11 | Yes  P:3 | Yes  P:3-4 | Yes  P:6-7 | Yes  P:3 | Yes  P:13-14 | Yes  P:5-6 | Yes  P:5 | Yes  P:4 |
| Measurement and valuation of resources and costs: Describe how costs were valued. | No | Yes  P:11 | Yes  P:11 | Yes  P:3 | Yes  P:3-4 | Yes  P:6 | Yes  P:3-4 | Yes  P:12-13 | Yes  P:6 | No | Yes  P:4 |
| Currency, price date, and conversion: Report the dates of the estimated resource quantities and unit costs, plus the currency and year of conversion. | Yes  P:16 | Yes  P:16 | Yes  P:16 | Yes  P:3 | Yes  P:5 | No | Yes  P:4 | Yes  P:12 | Yes  P:6 | No | Yes  P:4 |
| Rationale and description of model: If modelling is used, describe in detail, and why used. Report if the model is publicly available and where it can be accessed. | No | No | No | No | No | Yes  P:2-4 | No | No | No | No | Yes  P:3 |
| Analytics and assumptions: Describe any methods for analysing or statistically transforming data, any extrapolation methods, and approaches for validating any model used. | No | No | No | No | No | Yes  P:3 | Yes  P:3 | Yes  P:5 | Yes  P:2 | No | Yes  P:3 and appendix |
| Characterizing heterogeneity: Describe any methods used for estimating how the results of the study vary for subgroups. | Yes | No | No | No | No | No | No | No | Yes  P:7 | No | No |
| Characterizing distributional effects: Describe how impacts are distributed across different individuals or adjustments made to reﬂect priority populations. | No | No | No | No | No | No | No | No | No | No | No |
| Characterizing uncertainty: Describe methods to characterise any sources of uncertainty in the analysis. | No | No | No | No | No | Yes  P:7 | Yes  P:5-6 | Yes  P:14 | Yes  P:7 | Yes  P:6 | Yes  P:6 and 7 |
| Approach to engagement with patients and others affected by the study: Describe any approaches to engage patients or service recipients, the general public, communities, or stakeholders (such as clinicians or payers) in the design of the study. | Yes  P:10 | Yes  P:8-9 | Yes  P:9-10 | No | No | No | No | No | No | No | No |
| ***Results*** | | | | | | | | | | | |
| Study parameters: Report all analytic inputs (such as values, ranges, references) including uncertainty or distributional assumptions. | No | No | No | No | No | No | Yes  P:4 | Yes  P:4-10 | No | No | Yes  appendix |
| Summary of main results: Report the mean values for the main categories of costs and outcomes of interest and summarise them in the most appropriate overall measure. | Yes  P:12-13 | Yes  P:11 | Yes  P:12 | Yes  P:2 | Yes  P:7 | Yes  P:9 | Yes  P:6 | Yes  P:15 | Yes  P:7 | Yes  P:6 | Yes  P:7 and 8 |
| Effect of uncertainty: Describe how uncertainty about analytic judgments, inputs, or  projections affect ﬁndings. Report the effect of choice of discount rate and time horizon, if applicable. | Yes  P:12-13 | Yes  P:12 | Yes  P:12 | Yes  P:4 | Yes  P:8 | Yes  P:10-11 | Yes  P:6-7 | Yes  P:14-17 | Yes  P:8-9 | Yes  P:6 | Yes  P:6 |
| Effect of engagement with patients and others affected by the study: Report on any difference patient/service recipient, general public, community, or stakeholder involvement made to the approach or ﬁndings of the study | No | No | No | No | No | No | No | No | No | No | No |
| ***Discussion*** | | | | | | | | | | | |
| Study ﬁndings, limitations, generalizability, and current knowledge: Report key ﬁndings, limitations, ethical or equity considerations not captured, and how these could affect patients, policy, or practice. | Yes  P:12-13 | Yes  P:13 | Yes  P:12-13 | Yes  P:4-5 | Yes  P:9-11 | Yes  P:11-12 | Yes  P:6, 8-9 | Yes  P:15-18 | Yes  P:9-10 | No | Yes  P:8-10 |
| ***Other*** | | | | | | | | | | | |
| Source of Funding: Describe how the study was funded and any role of the funder in the identiﬁcation, design, conduct, and reporting of the analysis | No | No | No | Yes  P:5 | Yes  P:11 | Yes  P:13 | Yes  P:9 | Yes  P:18 | Yes  P:11 | No | Yes  P:11 |
| Conflicts of Interest: Report authors conﬂicts of interest according to journal or International Committee of Medical Journal Editors requirements. | No | No | No | Yes  P:5 | Yes  P:11 | Yes  P:12-13 | Yes  P:9 | Yes  P:18 | Yes  P:11 | No | Yes  P:11 |

**Appendix 5: Methodological quality assessment**

Table 6: Critical appraisal of the economic models using an adapted Philips checklist.[6]

| **Philips criteria** | | **NICE-Nusinersen TA588** | | **NICE-Risdiplam TA755** | **NICE-Onasemnogene HST15-type1** | | **NICE-Onasemnogene HST24-pre-symptomatic** | | **ICER Spinraza and zolgensma 2019** | **CADTH Nusinersen-2019** | **CADTH Risdiplam**  **2021** | **CADTH Onasemnogene -2021** | **Meijer et al.,**  **2023** | **Broekhoff et al., 2021** |
| --- | --- | --- | --- | --- | --- | --- | --- | --- | --- | --- | --- | --- | --- | --- |
| Structure | | | | | | | | | | | | | | |
| 1 | Is there a clear statement of the decision problem? | Yes | | Yes | Yes | | Yes | | Yes | Yes | Yes | Yes | Yes | Yes |
| 2 | Is the objective of the model specified and consistent with the stated decision problem? | Yes | | Yes | Yes | | Yes | | Yes | Yes | Yes | Yes | Yes | Yes |
| 3 | Is the primary decision maker specified? | Yes | | Yes | Yes | | Yes | | Yes | Yes | Yes | Yes | Unclear | Unclear |
| 4 | Is the perspective of the model stated clearly? | Yes | | Yes | Yes | | Yes | | Yes | Yes | Yes | Yes | Yes | Yes |
| 5 | Are the model inputs consistent with the stated perspective? | Yes | | Yes | Yes | | Yes | | Yes | Yes | Yes | Yes | Yes | Yes |
| 6 | Has the scope of the model been stated and justified? | Yes | | Yes | Yes | | Yes | | Yes | Yes | Yes | Yes | Unclear | Unclear |
| 7 | Are the outcomes of the model consistent with the perspective, scope and overall objective of the model? | Yes | | Yes | Yes | | Yes | | Yes | Yes | Yes | Yes | Yes | Yes |
| 8 | Is the structure of the model consistent with a coherent theory of the health condition under evaluation? | Yes | | Yes | Yes | | Yes | | Yes | Yes | Yes | Yes | Yes | No |
| 9 | Are the sources of the data used to develop the structure of the model specified? | Yes | | Yes | Yes | | Yes | | Yes | Yes | Yes | Yes | Yes | Yes |
| 10 | Are the causal relationships described by the model structure justified appropriately? | Yes | | Yes | Yes | | Yes | | Yes | Unclear | Yes | Yes | Yes | Yes |
| 11 | Are the structural assumptions transparent and justified? | Yes | | Yes | Yes | | Yes | | Yes | Yes | Yes | Yes | Yes | Yes |
| 12 | Are the structural assumptions reasonable given the overall objective, perspective and scope of the model? | Yes | | Yes | Yes | | Yes | | Yes | Yes | Yes | Yes | Yes | Yes |
| 13 | Is there a clear definition of the options under evaluation? | Yes | | Yes | Yes | | Yes | | Yes | Yes | Yes | Yes | Yes | Yes |
| 14 | Have all feasible and practical options been evaluated? | No | | No | No | | No | | No | No | No | No | No | No |
| 15 | Is there justification for the exclusion of feasible options? | Unclear | | Unclear | Unclear | | Unclear | | Unclear | Unclear | Unclear | Unclear | Unclear | Unclear |
| 16 | Is the chosen model type appropriate given the decision problem and specified casual relationships within the model? | Yes | | Yes | Yes | | Yes | | Yes | Yes | Yes | Yes | Yes | No |
| 17 | Is the time horizon of the model sufficient to reflect all important differences between the options? | Yes | | Yes | Yes | | Yes | | Yes | Yes | Yes | Yes | Yes | Yes |
| 18 | Are the time horizon of the model, the duration of treatment and the duration of treatment described and justified? | Yes | | Yes | Yes | | Yes | | Yes | Yes | Unclear | Yes | No | Yes |
| 19 | Do the disease states (state transition model) or the pathways (decision tree model) reflect the underlying biological process of the disease in question and the impact of interventions? | Yes | | Yes | Yes | | Yes | | Yes | Yes | Yes | Yes | Yes | No |
| 20 | Is the cycle length defined and justified in terms of the natural history of disease? | Yes | | Yes | Yes | | Yes | | Yes | Yes | Yes | Yes | Yes | Yes |
| DATA | |  |  | | |  | |  | |  |  |  |  | |
| 21 | Are the data identification methods transparent and appropriate given the objectives of the model? | Yes | | Unclear | Yes | | Yes | | Unclear | Unclear | Unclear | Unclear | Yes | Unclear |
| 22 | Where choices have been made between data sources are these justified appropriately? | Yes | | Yes | Yes | | Yes | | Yes | Unclear | Yes | Yes | Yes | Yes |
| 23 | Has particular attention been paid to identifying data for the important parameters of the model? | Yes | | Yes | Yes | | Yes | | Yes | Unclear | Yes | Yes | Yes | Yes |
| 24 | Has the quality of the data been assessed appropriately? | Yes | | Yes | Yes | | Yes | | Unclear | Unclear | Unclear | Unclear | Unclear | Unclear |
| 25 | Where expert opinion has been used are the methods described and justified? | Yes | | Yes | Unclear | | Unclear | | Yes | Yes | Unclear | Unclear | No | No |
| 26 | Is the data modelling methodology based on justifiable statistical and epidemiological techniques? | Yes | | Yes | Yes | | Yes | | Yes | Unclear | Yes | Yes | No | Unclear |
| 27 | Is the choice of baseline data described and justified? | Yes | | Yes | Yes | | Yes | | Yes | Yes | Yes | Yes | Yes | Yes |
| 28 | Are transition probabilities calculated appropriately? | Yes | | Yes | Yes | | Yes | | Yes | Unclear | Yes | Yes | No | Yes |
| 29 | Has a half-cycle correction been applied to both costs and outcomes? | Yes | | Yes | Yes | | Yes | | Yes | Unclear | Unclear | Unclear | Yes | Unclear |
| 30 | If not, has the omission been justified? | - | | - | - | | - | | - | Unclear | Unclear | Unclear | - | Unclear |
| 31 | If relative treatment effects have been derived from trial data, have they been synthesised using appropriate techniques? | Yes | | Yes | Yes | | Yes | | Yes | Yes | Yes | Unclear | Unclear | Unclear |
| 32 | Have the methods and assumptions used to extrapolate short-term results to final outcomes been documented and justified? | Yes | | Yes | Yes | | Yes | | Yes | Unclear | Yes | Yes | Yes | Yes |
| 33 | Have alternative extrapolation assumptions been explored through sensitivity analysis? | Yes | | Yes | Yes | | Yes | | Yes | Yes | Yes | Yes | Yes | Yes |
| 34 | Have assumptions regarding the continuing effect of treatment once treatment is complete been documented and justified? | Yes | | Yes | Yes | | Yes | | Yes | Yes | Yes | Yes | Yes | Yes |
| 35 | Have alternative assumptions regarding the continuing effect of treatment been explored through sensitivity analysis | No | | No | No | | No | | No | No | No | No | No | Yes |
| 36 | Are the costs incorporated into the model justified? | Yes | | Unclear | Yes | | Yes | | Yes | Yes | Yes | Yes | Yes | Yes |
| 37 | Has the source for all costs been described? | Yes | | Yes | Yes | | Yes | | Yes | Yes | Yes | Yes | Yes | Yes |
| 38 | Have discount rates been described and justified given the target decision maker? | Yes | | Yes | Yes | | Yes | | Yes | Yes | Yes | Yes | Yes | Yes |
| 39 | Are the utilities incorporated into the model appropriate? | Yes | | Yes | Yes | | Yes | | Yes | Unclear | Yes | Yes | Yes | Yes |
| 40 | Is the source of utility weights referenced? | Yes | | Yes | Yes | | Yes | | Yes | Yes | Yes | Yes | Yes | Yes |
| 41 | Are the methods of derivation for the utility weights justified? | Yes | | Yes | Yes | | Yes | | Unclear | Yes | Yes | Yes | Yes | Yes |
| 42 | Have all data incorporated into the model been described and referenced in sufficient detail? | Yes | | Yes | Yes | | Yes | | Yes | Unclear | Unclear | Unclear | Unclear | Unclear |
| 43 | Has the use of mutually inconsistent data been justified (i.e. are assumptions and choices appropriate?) | Yes | | Yes | Yes | | Yes | | Yes | Yes | Yes | Yes | Unclear | Unclear |
| 44 | Is the process of data incorporation transparent? | Yes | | Yes | Yes | | Yes | | Yes | Yes | Yes | Yes | Yes | Yes |
| 45 | If data have been incorporated as distributions, has the choice of distributions for each parameter been described and justified? | Yes | | Unclear | Yes | | Yes | | Yes | Unclear | Unclear | Unclear | Yes | Yes |
| 46 | If data have been incorporated as distributions, is it clear that second order uncertainty is reflected? | Yes | | Unclear | Yes | | Yes | | Yes | Unclear | Unclear | Unclear | Yes | Yes |
| 47 | Have the four principal types of uncertainty been addressed? | Yes | | Yes | Yes | | Yes | | Yes | No | No | No | No | No |
| 48 | If not, has the omission of particular forms of uncertainty been justified? | - | | - | - | | - | | - | No | No | No | No | No |
| 49 | Have methodological uncertainties been addressed by running alternative versions of the model with different methodological assumptions? | Yes | | Yes | Yes | | Yes | | Yes | Yes | Yes | Yes | Yes | Yes |
| 50 | Is there evidence that structural uncertainties have been addressed via sensitivity analysis? | Yes | | Yes | Yes | | Yes | | Yes | Yes | Yes | Yes | Yes | Yes |
| 51 | Has heterogeneity been dealt with by running the model separately for different sub-groups? | Yes | | Yes | Yes | | Yes | | Yes | Yes | Yes | Yes | Yes | No |
| 52 | Are the methods of assessment of parameter uncertainty appropriate? | Yes | | Yes | Yes | | Yes | | Yes | Unclear | Yes | Yes | Yes | Yes |
| 53 | If data are incorporated as point estimates, are the ranges used for sensitivity analysis stated clearly and justified? | Yes | | Yes | Yes | | Yes | | Yes | No | No | No | Yes | Yes |
| 54 | Is there evidence that the mathematical logic of the model has been tested thoroughly before use? | Unclear | | Unclear | Unclear | | Unclear | | Unclear | Unclear | Unclear | Unclear | Unclear | Unclear |
| 55 | Are any counterintuitive results from the model explained and justified? | Yes | | Yes | Yes | | Yes | | Yes | Yes | Yes | Yes | Yes | Yes |
| 56 | If the model has been calibrated against independent data, have any differences been explained and justified? | Unclear | | Unclear | Unclear | | - | | - | - | - | - | - | - |
| 57 | Have the results been compared with those of previous models and any differences in results explained? | Yes | | Yes | Yes | | Yes | | Unclear | No | No | No | Yes | Yes |
| N- No; N/A- Not Applicable; Y- Yes; UNC-Unclear | | | | | | | | | | | | | | |

Table 2: Critical appraisal of the economic models using the Philips checklist (cont’d)

| **Philips criteria** | | **SMC Nusinersen- 2018** | **SMC Onasemnogene -2021** | | **SMC Risdiplam-2022** | | **Connock et al., 2020** | | **Dean et al. 2021** | **Malone et al. 2019** | **Wang et al. 2022** | **Zuluaga‑Sanchez et al. 2019** | **Thokala et al. 2020** | **NCPE-2017** | **Khuntha S, et al., 2025** |
| --- | --- | --- | --- | --- | --- | --- | --- | --- | --- | --- | --- | --- | --- | --- | --- |
| Structure | | | | | | | | | | | | | | |  |
| 1 | Is there a clear statement of the decision problem? | Unclear | Unclear | | Unclear | | Yes | | Yes | Yes | Yes | Yes | Yes | Yes | Yes |
| 2 | Is the objective of the model specified and consistent with the stated decision problem? | Yes | Yes | | Yes | | Yes | | Yes | Yes | Yes | Yes | Yes | Yes | Yes |
| 3 | Is the primary decision maker specified? | Yes | Yes | | Yes | | Yes | | Yes | Yes | Yes | Yes | Yes | Yes | Yes |
| 4 | Is the perspective of the model stated clearly? | Yes | Yes | | Yes | | Yes | | Yes | Yes | Yes | Yes | Yes | Yes | Yes |
| 5 | Are the model inputs consistent with the stated perspective? | Yes | Yes | | Yes | | Yes | | Yes | Yes | Yes | Yes | Yes | Yes | Yes |
| 6 | Has the scope of the model been stated and justified? | Unclear | Unclear | | Unclear | | Unclear | | Unclear | Unclear | Unclear | Unclear | Unclear | Unclear | Unclear |
| 7 | Are the outcomes of the model consistent with the perspective, scope and overall objective of the model? | Yes | Yes | | Yes | | Yes | | Yes | Yes | Yes | Yes | Yes | Yes | Yes |
| 8 | Is the structure of the model consistent with a coherent theory of the health condition under evaluation? | Yes | Yes | | Yes | | No | | Yes | Yes | Yes | Yes | Yes | Unclear | Unclear |
| 9 | Are the sources of the data used to develop the structure of the model specified? | Yes | Yes | | Yes | | Yes | | Yes | Yes | Yes | Yes | Yes | Yes | Yes |
| 10 | Are the causal relationships described by the model structure justified appropriately? | Yes | Yes | | Yes | | Unclear | | Yes | Yes | Yes | Yes | Yes | Unclear | Yes |
| 11 | Are the structural assumptions transparent and justified? | No | No | | No | | Unclear | | Yes | Yes | Yes | Yes | Yes | Unclear | Yes |
| 12 | Are the structural assumptions reasonable given the overall objective, perspective and scope of the model? | No | No | | No | | Unclear | | Unclear | Yes | Yes | Yes | Yes | Unclear | Yes |
| 13 | Is there a clear definition of the options under evaluation? | Yes | Yes | | Yes | | Yes | | Yes | Yes | Yes | Yes | Yes | Yes | Yes |
| 14 | Have all feasible and practical options been evaluated? | Yes | No | | No | | Yes | | Yes | Yes | No | Yes | No | Yes | No |
| 15 | Is there justification for the exclusion of feasible options? | - | Unclear | | Unclear | | - | | - | - | Unclear | - | Unclear | - | Unclear |
| 16 | Is the chosen model type appropriate given the decision problem and specified casual relationships within the model? | Yes | Yes | | Yes | | Unclear | | Yes | Yes | Yes | Yes | Yes | Unclear | Yes |
| 17 | Is the time horizon of the model sufficient to reflect all important differences between the options? | Yes | Yes | | Yes | | Yes | | Yes | Yes | Yes | Yes | Yes | Yes | Yes |
| 18 | Are the time horizon of the model, the duration of treatment and the duration of treatment described and justified? | Yes | Yes | | Yes | | No | | Yes | Yes | Yes | Yes | Yes | Unclear | Yes |
| 19 | Do the disease states (state transition model) or the pathways (decision tree model) reflect the underlying biological process of the disease in question and the impact of interventions? | Yes | Yes | | Yes | | No | | Yes | Yes | Yes | Yes | Yes | Unclear | Yes |
| 20 | Is the cycle length defined and justified in terms of the natural history of disease? | Unclear | Yes | | Yes | | No | | Unclear | Yes | Yes | Yes | Yes | Yes | Yes |
| Data | |  | |  | |  | |  | |  |  |  |  | |  |
| 21 | Are the data identification methods transparent and appropriate given the objectives of the model? | Yes | Yes | | Yes | | Yes | | Yes | Yes | Yes | Yes | Yes | Unclear | Yes |
| 22 | Where choices have been made between data sources are these justified appropriately? | Yes | Yes | | Yes | | Yes | | Yes | Yes | Yes | Yes | Yes | Unclear | Yes |
| 23 | Has particular attention been paid to identifying data for the important parameters of the model? | Yes | Yes | | Yes | | Yes | | Yes | Yes | Yes | Yes | Yes | Unclear | Yes |
| 24 | Has the quality of the data been assessed appropriately? | Unclear | Unclear | | Unclear | | Unclear | | Unclear | Unclear | Unclear | Unclear | Unclear | Unclear | Unclear |
| 25 | Where expert opinion has been used are the methods described and justified? | Unclear | Unclear | | Unclear | | Unclear | | Unclear | Unclear | Unclear | Unclear | Unclear | Unclear | Unclear |
| 26 | Is the data modelling methodology based on justifiable statistical and epidemiological techniques? | Unclear | Unclear | | Unclear | | Unclear | | Unclear | Unclear | Unclear | Unclear | Unclear | Unclear | Unclear |
| 27 | Is the choice of baseline data described and justified? | Unclear | Unclear | | Unclear | | Yes | | Unclear | Yes | Yes | Yes | Yes | Unclear | Yes |
| 28 | Are transition probabilities calculated appropriately? | Unclear | Unclear | | Yes | | Unclear | | Unclear | Yes | Yes | Yes | Yes | Unclear | Yes |
| 29 | Has a half-cycle correction been applied to both costs and outcomes? | Unclear | Unclear | | Unclear | | Unclear | | Unclear | Unclear | Yes | Yes | Unclear | Unclear | Unclear |
| 30 | If not, has the omission been justified? | - | - | | - | | - | | - | - | - | - | - | - | - |
| 31 | If relative treatment effects have been derived from trial data, have they been synthesised using appropriate techniques? | Unclear | Unclear | | Yes | | Unclear | | Unclear | Unclear | Unclear | Unclear | Unclear | Unclear | Unclear |
| 32 | Have the methods and assumptions used to extrapolate short-term results to final outcomes been documented and justified? | Unclear | Unclear | | Unclear | | Unclear | | Yes | Yes | Yes | Yes | Yes | Unclear | Yes |
| 33 | Have alternative extrapolation assumptions been explored through sensitivity analysis? | Unclear | Unclear | | Unclear | | No | | Unclear | Unclear | Unclear | Unclear | Unclear | Unclear | Unclear |
| 34 | Have assumptions regarding the continuing effect of treatment once treatment is complete been documented and justified? | Yes | Yes | | Yes | | Unclear | | Unclear | Yes | Unclear | Yes | Unclear | Unclear | Unclear |
| 35 | Have alternative assumptions regarding the continuing effect of treatment been explored through sensitivity analysis | Unclear | Unclear | | Unclear | | Unclear | | Unclear | Unclear | Unclear | Unclear | Unclear | Unclear | Unclear |
| 36 | Are the costs incorporated into the model justified? | Unclear | Yes | | Unclear | | Yes | | Yes | Yes | Yes | Yes | Yes | Unclear | Yes |
| 37 | Has the source for all costs been described? | Unclear | Yes | | Yes | | Yes | | Yes | Yes | Yes | Yes | Yes | Unclear | Yes |
| 38 | Have discount rates been described and justified given the target decision maker? | Unclear | Unclear | | Unclear | | Unclear | | Yes | Yes | Yes | Yes | Yes | Yes | Yes |
| 39 | Are the utilities incorporated into the model appropriate? | Yes | No | | No | | Yes | | Yes | Yes | Yes | Yes | Yes | Unclear | Yes |
| 40 | Is the source of utility weights referenced? | Yes | Yes | | Yes | | Yes | | Yes | Yes | Yes | Yes | Yes | Yes | Yes |
| 41 | Are the methods of derivation for the utility weights justified? | Unclear | Unclear | | Unclear | | Unclear | | Yes | Yes | Unclear | Yes | Yes | Yes | Yes |
| 42 | Have all data incorporated into the model been described and referenced in sufficient detail? | No | No | | No | | Unclear | | Unclear | Yes | Yes | Yes | Yes | Unclear | Yes |
| 43 | Has the use of mutually inconsistent data been justified (i.e. are assumptions and choices appropriate?) | No | No | | No | | Yes | | No | No | No | No | No | Unclear | Unclear |
| 44 | Is the process of data incorporation transparent? | Unclear | Unclear | | Unclear | | Yes | | Unclear | Yes | Yes | Yes | Yes | Unclear | Yes |
| 45 | If data have been incorporated as distributions, has the choice of distributions for each parameter been described and justified? | Unclear | Unclear | | Unclear | | Unclear | | Unclear | Unclear | Yes | Yes | Unclear | Unclear | Yes |
| 46 | If data have been incorporated as distributions, is it clear that second order uncertainty is reflected? | Unclear | Unclear | | Unclear | | Unclear | | Unclear | Unclear | Unclear | Yes | Unclear | Unclear | Unclear |
| 47 | Have the four principal types of uncertainty been addressed? | Unclear | Unclear | | Unclear | | No | | No | Yes | Yes | Yes | Yes | Unclear | Yes |
| 48 | If not, has the omission of particular forms of uncertainty been justified? | - | - | | - | | Unclear | | No | - | - | - | - | Unclear | - |
| 49 | Have methodological uncertainties been addressed by running alternative versions of the model with different methodological assumptions? | Yes | Yes | | Yes | | Yes | | Yes | Yes | Yes | Yes | Yes | Yes | Yes |
| 50 | Is there evidence that structural uncertainties have been addressed via sensitivity analysis? | Unclear | Unclear | | Unclear | | Yes | | Yes | Unclear | Unclear | Unclear | Unclear | Unclear | Unclear |
| 51 | Has heterogeneity been dealt with by running the model separately for different sub-groups? | Unclear | Unclear | | Unclear | | No | | No | No | No | Yes | No | Unclear |  |
| 52 | Are the methods of assessment of parameter uncertainty appropriate? | Yes | Yes | | Yes | | No | | Yes | Yes | Yes | Yes | Yes | Unclear | Yes |
| 53 | If data are incorporated as point estimates, are the ranges used for sensitivity analysis stated clearly and justified? | Unclear | Unclear | | Unclear | | Unclear | | No | No | Yes | Yes | Yes | Unclear | Yes |
| 54 | Is there evidence that the mathematical logic of the model has been tested thoroughly before use? | Unclear | Unclear | | Unclear | | Unclear | | Unclear | Unclear | Unclear | Unclear | Unclear | Unclear | Unclear |
| 55 | Are any counterintuitive results from the model explained and justified? | No | No | | No | | Yes | | Yes | Yes | Yes | Yes | Yes | No | Yes |
| 56 | If the model has been calibrated against independent data, have any differences been explained and justified? | Unclear | Unclear | | Unclear | | Unclear | | Unclear | Unclear | Unclear | Unclear | Unclear | Unclear | Yes |
| 57 | Have the results been compared with those of previous models and any differences in results explained? | No | No | | No | | Yes | | Yes | Yes | Yes | Yes | Yes | No | Yes |
| N- No; N/A- Not Applicable; Y- Yes; UNC-Unclear | | | | | | | | | | | | | | |  |

**References**

1. Paracha N, Hudson P, Mitchell S, Sutherland CS. Systematic literature review to assess economic evaluations in spinal muscular atrophy (SMA). Pharmacoeconomics. 2022;40(Suppl 1):69-89.

2. Wiedmann L, Cairns J. Review of economic modeling evidence from NICE appraisals of rare disease treatments for spinal muscular atrophy. Expert Rev Pharmacoecon Outcomes Res. 2023;23(5):469-82.

3. Yang M, Awano H, Tanaka S, Toro W, Zhang S, Dabbous O, et al. Systematic literature review of clinical and economic evidence for spinal muscular atrophy. Adv Ther. 2022;39(5):1915-58.

4. Dangouloff T, Botty C, Beaudart C, Servais L, Hiligsmann M. Systematic literature review of the economic burden of spinal muscular atrophy and economic evaluations of treatments. Orphanet J Rare Dis. 2021;16(1):47.

5. Husereau D, Drummond M, Augustovski F, de Bekker-Grob E, Briggs AH, Carswell C, et al. Consolidated Health Economic Evaluation Reporting Standards 2022 (CHEERS 2022) statement: updated reporting guidance for health economic evaluations. Value Health. 2022;25(1):3-9.

6. Philips Z, Ginnelly L, Sculpher M, Claxton K, Golder S, Riemsma R, et al. Review of guidelines for good practice in decision-analytic modelling in health technology assessment. Health Technol Assess. 2004;8(36):1-158.
